# Development and internal validation of machine-learning models for postoperative deep vein thrombosis after hip arthroplasty for femoral neck fracture: evaluation of Traditional Chinese Medicine syndrome features

**DOI:** 10.3389/frai.2026.1798230

**Published:** 2026-10-29

**Authors:** Yang Liu, Zhaoke Pi, Yuwei Shen, Peilian Cao, Yuqiang Qian, Daxing Jiang, Jianan Li, Peng Yuan

**Affiliations:** 1 Nanjing Medical University, Nanjing, Jiangsu, China; 2 National-Regional Key Technology Engineering Laboratory for Medical Ultrasound, Guangdong Key Laboratory for Biomedical Measurements and Ultrasound Imaging, School of Biomedical Engineering, Shenzhen University Medical School, Shenzhen University, Shenzhen, China; 3 Department of Rehabilitation Medicine Center, West China Hospital, Chengdu, Sichuan, China; 4 Gangcheng Rehabilitation Hospital, Zhangjiagang Aoyang Hospital, Zhangjiagang, Jiangsu, China

**Keywords:** deep vein thrombosis, femoral neck fracture, hip arthroplasty, machine learning, SHAP, Traditional Chinese Medicine, TyG index

## Abstract

**Background:**

Postoperative lower-extremity deep vein thrombosis (DVT) remains a clinically important complication after hip arthroplasty for femoral neck fracture. We evaluated whether Traditional Chinese Medicine (TCM) syndrome features add predictive information beyond conventional preoperative variables and examined the association between the triglyceride-glucose (TyG) index and postoperative DVT.

**Methods:**

This retrospective single-center study included 380 patients. Group-constrained partitioning and resampling prevented identical prespecified multivariable profiles from crossing evaluation boundaries, yielding a development set (*n* = 267; 74 DVT events) and a profile-disjoint held-out validation set (*n* = 113; 31 events). Nine machine-learning algorithms were evaluated using a fixed eight-variable panel comprising D-dimer, fibrinogen, platelet count, type 2 diabetes, fasting blood glucose, TyG, Qi stagnation with blood stasis (QSBS), and Qi deficiency with blood stasis (QDBS). Random forest (RF) was selected on the basis of development-set out-of-fold performance before held-out validation.

**Results:**

Postoperative DVT occurred in 105 patients (27.6%). RF achieved a pooled group-aware development out-of-fold AUC of 0.774 (95% CI 0.710–0.837) and a held-out validation AUC of 0.859 (95% CI 0.782–0.935). Across 30 repeated group-constrained partitions, mean validation AUC was 0.792. In the single profile-disjoint held-out validation partition, the development-derived threshold of 0.298 yielded sensitivity of 0.903, specificity of 0.720, positive predictive value of 0.549, and negative predictive value of 0.952; in that same partition, the calibration slope was 1.136 (95% CI 0.753–1.867), the observed-to-expected ratio was 0.846, and the Brier score was 0.132. Adding QSBS and QDBS did not materially improve discrimination (AUC 0.855 vs. 0.859; ΔAUC = 0.003; DeLong *p* = 0.746). Higher TyG remained associated with DVT after multivariable adjustment (OR per 1 SD 1.550, 95% CI 1.186–2.026; *p* = 0.001); restricted cubic splines supported an overall association (*p* = 0.007) without significant evidence of nonlinearity (*p* = 0.082).

**Conclusion:**

RF showed favorable discrimination in the held-out cohort but more modest performance across repeated group-constrained partitions. The two retained TCM syndrome variables did not provide a material increment in AUC, whereas higher TyG remained associated with postoperative DVT after multivariable adjustment. External validation and assessment of recalibration are required before clinical implementation.

## 1 Introduction

Postoperative deep vein thrombosis (DVT) is an important complication after hip-fracture surgery and major joint arthroplasty. Risk is influenced by patient comorbidity, delayed surgery, immobility, and other perioperative factors, and contemporary guidelines recommend thromboprophylaxis for patients with hip fracture (Ngarmukos et al., 2021; American Academy of Orthopaedic Surgeons, 2021). Observational studies nevertheless show substantial heterogeneity in perioperative DVT risk among patients with femoral neck fracture (Wang et al., 2022; Song et al., 2016; Fu et al., 2020; Dou et al., 2022), supporting the need for population-specific risk stratification.

Existing risk-assessment approaches do not perform uniformly across orthopedic populations. The Caprini score, for example, has shown limited discrimination or limited treatment-guiding utility in some total joint arthroplasty cohorts (Gibbs et al., 2023; Gold et al., 2020), while procedure-specific prediction tools have been developed for postoperative DVT after femoral fracture surgery (Wu and Cheng, 2023). These differences emphasize that prediction models should be evaluated in the population and clinical setting in which they are intended to be used.

Machine learning (ML) can model nonlinear associations and interactions among clinical predictors, but increased algorithmic flexibility does not necessarily translate into better clinical prediction. Comparative evidence has not shown a consistent advantage of ML over logistic regression in studies at low risk of bias, and incomplete internal validation and calibration assessment remain common concerns (Rajkomar et al., 2019; Christodoulou et al., 2019; Wynants et al., 2017). ML models have nevertheless shown promise for DVT prediction in orthopedic populations when model development, validation, and reporting are conducted transparently (Wei et al., 2024; Wu et al., 2024; Huang et al., 2025).

Traditional Chinese Medicine (TCM) syndrome differentiation integrates constellations of symptoms, signs, tongue findings, and pulse findings rather than isolated variables (Jiang et al., 2012). In the present cohort, seven preoperative syndrome labels were available, including Qi deficiency with blood stasis (QDBS) and Qi stagnation with blood stasis (QSBS). Whether these syndrome features contain incremental predictive information beyond routinely available clinical and laboratory variables has not been established and requires direct empirical evaluation.

The triglyceride-glucose (TyG) index is a practical surrogate marker of insulin resistance derived from fasting triglyceride and glucose concentrations (Guerrero-Romero et al., 2010; Yin et al., 2024). Evidence linking TyG to venous thrombosis is emerging but heterogeneous: a study in total knee arthroplasty reported a positive association with postoperative DVT, whereas a UK Biobank analysis found no independent association between TyG alone and incident venous thromboembolism (Wu et al., 2025; Wang et al., 2026). Its relationship with postoperative DVT after hip arthroplasty for femoral neck fracture therefore remains clinically relevant but uncertain.

Accordingly, this study had three objectives: (1) to develop and internally validate nine ML models using preoperative clinical, laboratory, and TCM features; (2) to quantify the incremental predictive value of QDBS and QSBS beyond a clinical/laboratory model; and (3) to examine the multivariable-adjusted and potentially nonlinear association between continuous TyG and postoperative lower-extremity DVT.

## 2 Methods

### 2.1 Study design and population

This retrospective single-center study assessed consecutive adults aged ≥18 years with radiographically confirmed femoral neck fracture who underwent hip arthroplasty during the index hospitalization between October 2023 and June 2025. Patients treated with internal fixation were outside the arthroplasty screening frame and were therefore not included among the 481 patients assessed for eligibility. Exclusion criteria were preoperative venous thromboembolism (VTE; *n* = 31), pre-admission anticoagulant use (*n* = 22), and missing key baseline variables required for analysis (*n* = 48). Of 481 patients assessed, 380 remained in the analytical cohort (Figure 1). Arthroplasty subtype was not available as a validated analytical variable and was therefore not analyzed.

**Figure 1:**
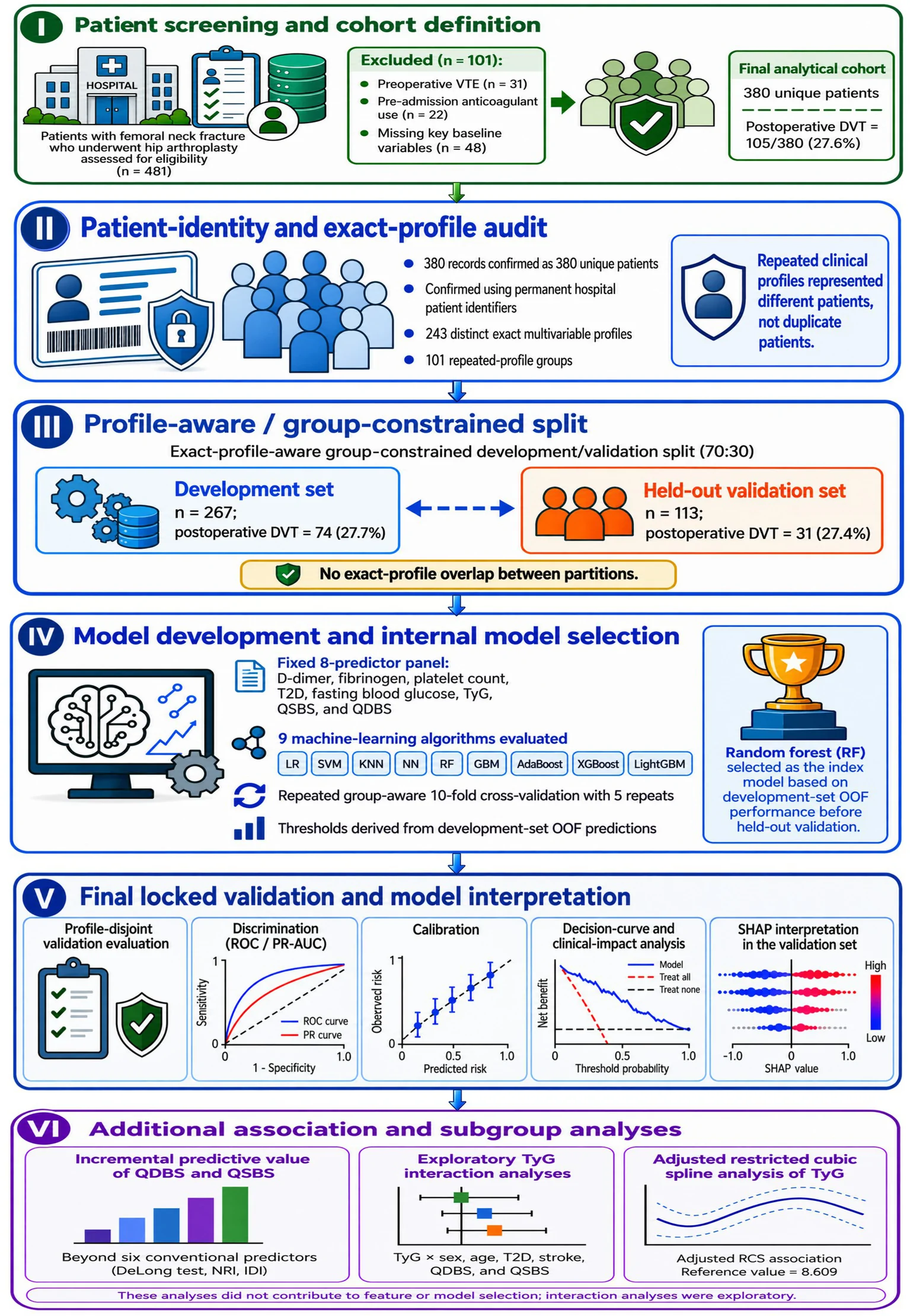
Study workflow and analytical pipeline. Among 481 patients with femoral neck fracture who underwent hip arthroplasty and were assessed for eligibility, 380 unique patients entered the final analytical cohort after prespecified exclusions. Profile-aware group-constrained procedures were then used for model development and held-out validation. Random forest was selected from development-set out-of-fold performance; downstream analyses assessed TCM incremental value, TyG associations, and exploratory TyG interactions.

Each analytical record corresponded to a unique patient, confirmed during cohort assembly using permanent hospital identifiers; all direct identifiers were removed before analysis.

The study was reported in accordance with the TRIPOD+AI Statement (Collins et al., 2024).

### 2.2 Data collection

Baseline variables included sex, age, height, weight, body mass index (BMI), hypertension, type 2 diabetes (T2D), heart disease, prior stroke, dyslipidemia, injury-to-surgery interval, and perioperative immobilization duration. Laboratory variables included prothrombin time (PT), international normalized ratio (INR), activated partial thromboplastin time (APTT), fibrinogen, D-dimer, C-reactive protein (CRP), fasting blood glucose (FBG), triglycerides (TG), total cholesterol (TC), high-density lipoprotein cholesterol (HDL-C), low-density lipoprotein cholesterol (LDL-C), white blood cell count, neutrophils, lymphocytes, red blood cell count, hemoglobin, and platelet count. Laboratory predictors were defined as the first available measurements after admission and before surgery. BMI was calculated from recorded height and weight.

TG and FBG were recorded in mmol/L and converted to mg/dL for calculation of TyG: TyG = ln{[TG (mmol/L) × 88.57] × [FBG (mmol/L) × 18.0]/2}. TyG was recalculated from the recorded TG and FBG values for every patient using this conventional formula.

### 2.3 TCM syndrome differentiation

TCM syndrome assessment was performed after admission and before surgery using the contemporaneous preoperative TCM record, including documented symptoms and signs and clinician-recorded tongue and pulse findings. Two senior TCM physicians, each with more than 10 years of clinical experience, assessed seven non-mutually exclusive binary syndromes using prespecified definitions aligned with the Chinese national standard GB/T 16751.2–2021 (State Administration for Market Regulation, Standardization Administration of China, 2021): QDBS, QSBS, Yin deficiency and blood stasis (YDBS), cold coagulation with blood stasis (CCBS), dampness-heat and blood stasis obstruction (DHBSO), phlegm-blood stasis binding (PBSB), and heat-toxin and blood stasis (HTBS). Disagreements were resolved by consensus, and the consensus labels were used for analysis.

### 2.4 Outcome definition

The outcome was postoperative lower-extremity DVT diagnosed during hospitalization. All included patients underwent routine postoperative lower-extremity venous duplex ultrasonography during hospitalization; patients without ultrasound-confirmed DVT were classified as non-DVT. Pulmonary embolism was not included in the modeled outcome.

### 2.5 ML modeling

To prevent the same multivariable predictor pattern from appearing in both development and validation data, records sharing an identical 23-variable demographic, clinical, and laboratory profile (listed in Supplementary Methods) were treated as a group. Group-constrained partitioning assigned the cohort approximately 70:30 to a development set (*n* = 267; 74 DVT events) and a held-out validation set (*n* = 113; 31 events), with no exact-profile overlap between partitions.

The same grouping constraint was applied during development-set resampling so that members of an exact profile remained within the same fold. Nine algorithms were evaluated: logistic regression (LR), support vector machine (SVM), gradient boosting machine (GBM), neural network (NN), random forest (RF), XGBoost (XGB), k-nearest neighbors (KNN), AdaBoost, and LightGBM (LGBM) (Cortes and Vapnik, 1995; Friedman, 2001; Breiman, 2001; Chen and Guestrin, 2016; Freund and Schapire, 1997; Ke et al., 2017). Hyperparameters were tuned using repeated 10-fold cross-validation with five repeats. Classification thresholds were derived exclusively from pooled development-set out-of-fold (OOF) predictions using Youden’s index and were applied unchanged to the held-out validation set. The primary analysis preserved the observed class distribution; fold-nested SMOTENC was examined as a sensitivity analysis (Chawla et al., 2002).

The predictor panel was specified using development data only. TyG, QDBS, and QSBS were retained *a priori* to address the metabolic and TCM study questions. Among the remaining clinical/laboratory candidates with a development-set likelihood-ratio-test *p* < 0.05, five variables were selected in ascending order of *p*-value to complete an eight-predictor panel while keeping model complexity proportionate to the 74 development events, with reference to contemporary guidance on sample size and model complexity for binary prediction models (Riley et al., 2019). The resulting panel comprised D-dimer, fibrinogen, platelet count, T2D, FBG, TyG, QSBS, and QDBS. Candidate screening and panel specification were performed once in the development set before held-out validation; no validation-set information was used.

RF was selected as the index model on the basis of development-set OOF AUC before the held-out validation set was evaluated. The remaining eight algorithms were evaluated using the same fixed predictor panel and group-constrained data structure as comparators.

Continuous predictors were centered and scaled within resampling. For KNN, principal component analysis was additionally performed within resampling to retain approximately 95% of the variance. Median imputation was prespecified for missing continuous values but was not required because the retained analytical variables were complete (Supplementary Table S9).

### 2.6 Model evaluation

Model performance was evaluated in the profile-disjoint held-out validation set. Discrimination was summarized by the area under the receiver operating characteristic curve (AUC) and precision-recall AUC (PR-AUC) (Saito and Rehmsmeier, 2015). Threshold-based performance included sensitivity, specificity, accuracy, positive predictive value (PPV), negative predictive value (NPV), and F1-score. Calibration was assessed using the calibration intercept and slope, observed-to-expected (O: E) ratio, Brier score, scaled Brier score, and calibration plots (Van Calster et al., 2019). For the RF index model, the 95% confidence interval for the calibration slope was estimated using 5,000 patient-level nonparametric bootstrap resamples of the held-out validation cohort, with percentile-based confidence limits. Clinical utility was explored using decision-curve and clinical-impact analyses (Vickers and Elkin, 2006).

The incremental value of QDBS and QSBS was evaluated by comparing otherwise matched six-variable clinical/laboratory and eight-variable integrated RF models. Comparisons included change in AUC with paired DeLong testing (DeLong et al., 1988), calibration measures, scaled Brier score, continuous net reclassification improvement (NRI), and integrated discrimination improvement (IDI). TCM collinearity diagnostics are reported in Supplementary Table S3.

Sensitivity of RF performance to the development-validation partition was assessed across 30 repeated group-constrained 70:30 splits, with all members of each exact profile kept together. Held-out AUCs were summarized by the mean, median, standard deviation, and 2.5th–97.5th percentiles. These repeated partitions were used to characterize internal stability and did not contribute to feature or model selection, consistent with established guidance on internal validation and prediction-model evaluation (Steyerberg et al., 2001; Collins et al., 2024; Supplementary Table S6).

### 2.7 Model interpretability

SHapley Additive exPlanations (SHAP) (Lundberg and Lee, 2017; Lundberg et al., 2020) were calculated for the eight-variable RF index model in the profile-disjoint validation set (*n* = 113). Global importance was summarized by mean absolute SHAP values, and summary and dependence plots were used to display the magnitude and direction of feature contributions. SHAP values were interpreted as model-specific predictive contributions rather than causal effects.

### 2.8 TyG association and exploratory interaction analyses

TyG was analyzed continuously in the primary association analysis. Logistic regression estimated odds ratios (ORs) per 1-unit and per 1-standard-deviation (SD) increase in TyG, both unadjusted and multivariable-adjusted. The adjustment set comprised age and sex (demographic factors), T2D (metabolic comorbidity), D-dimer and fibrinogen (hemostatic markers), and QDBS and QSBS (the two TCM syndrome variables evaluated in the integrated prediction model). A secondary dichotomized analysis used the development-set median TyG of 8.609.

The adjusted restricted cubic spline (RCS) model used four knots at TyG values of 7.938, 8.437, 8.781, and 9.678, with 8.609 as the reference value and the same covariate adjustment.

Exploratory effect-modification analyses were conducted in the full analytical cohort, separately from prediction-model development and validation. Interactions of TyG with sex, age group, T2D, prior stroke, QDBS, and QSBS were evaluated using likelihood-ratio tests; the age-group cutoff was the development-set median age of 79 years. Each interaction model included TyG (per 1 SD), the subgroup variable, and their interaction term without additional covariate adjustment. Benjamini–Hochberg false-discovery-rate (FDR) correction was applied across the six interaction tests. These analyses were not used for feature selection, tuning, model selection, or validation.

### 2.9 Statistical analysis and software

Continuous variables were summarized as mean ± standard deviation (SD) or median [interquartile range (IQR)], as appropriate. Baseline continuous variables were compared using the Mann–Whitney U test and categorical variables using Fisher’s exact test. Statistical tests were two-sided, with *p* < 0.05 considered statistically significant unless otherwise specified; Benjamini–Hochberg FDR-adjusted *p-*values were used for the six interaction tests.

Final reproducibility verification was performed in R version 4.6.1. The current R and package environment was documented using the R sessionInfo function; model engines, hyperparameters, preprocessing specifications, random seeds, and group-constrained resampling procedures are reported in Supplementary Table S10.

## 3 Results

### 3.1 Cohort and baseline characteristics

Of 481 patients with radiographically confirmed femoral neck fracture who underwent hip arthroplasty and were assessed for eligibility, 101 were excluded: 31 for preoperative VTE, 22 for pre-admission anticoagulant use, and 48 for missing key baseline variables. The final analytical cohort comprised 380 patients, of whom 105 (27.6%) developed postoperative lower-extremity DVT.

Compared with patients without DVT, those with postoperative DVT were more frequently female (81.9% vs. 66.2%, *p* = 0.002) and more often had hypertension (64.8% vs. 50.2%, *p* = 0.011), T2D (40.0% vs. 20.7%, *p* < 0.001), and prior stroke (32.4% vs. 15.6%, *p* = 0.001). QDBS (54.3% vs. 41.5%, *p* = 0.028) and QSBS (48.6% vs. 36.7%, *p* = 0.046) were also more frequent in the DVT group.

Patients with DVT had a longer injury-to-surgery interval (median 168 vs. 124 h, *p* = 0.034), higher fibrinogen, D-dimer, CRP, FBG, TG, corrected TyG, LDL-C, and platelet count, and lower HDL-C and hemoglobin (all *p* < 0.05) (Table 1). Median corrected TyG was 8.77 in patients with DVT and 8.54 in those without DVT (*p* = 0.001).

**Table 1:** Baseline characteristics of patients with and without postoperative lower-extremity DVT.

Variable	Overall (*n* = 380)	Non-DVT (*n* = 275)	DVT (*n* = 105)	*p-*value	SMD
*n*	380	275	105		
Sex, *n* (%): Female	268 (70.5)	182 (66.2)	86 (81.9)	0.002	0.365
Male	112 (29.5)	93 (33.8)	19 (18.1)		
Hypertension, *n* (%): No	174 (45.8)	137 (49.8)	37 (35.2)	0.011	0.298
Yes	206 (54.2)	138 (50.2)	68 (64.8)		
Type 2 diabetes, *n* (%): No	281 (73.9)	218 (79.3)	63 (60.0)	<0.001	0.429
Yes	99 (26.1)	57 (20.7)	42 (40.0)		
Prior stroke, *n* (%): No	303 (79.7)	232 (84.4)	71 (67.6)	0.001	0.400
Yes	77 (20.3)	43 (15.6)	34 (32.4)		
Heart disease, *n* (%): No	323 (85.0)	233 (84.7)	90 (85.7)	0.874	0.028
Yes	57 (15.0)	42 (15.3)	15 (14.3)		
Dyslipidemia, *n* (%): No	338 (88.9)	247 (89.8)	91 (86.7)	0.367	0.098
Yes	42 (11.1)	28 (10.2)	14 (13.3)		
QDBS, *n* (%): No	209 (55.0)	161 (58.5)	48 (45.7)	0.028	0.259
Yes	171 (45.0)	114 (41.5)	57 (54.3)		
QSBS, *n* (%): No	228 (60.0)	174 (63.3)	54 (51.4)	0.046	0.241
Yes	152 (40.0)	101 (36.7)	51 (48.6)		
CCBS, *n* (%): No	286 (75.3)	208 (75.6)	78 (74.3)	0.791	0.031
Yes	94 (24.7)	67 (24.4)	27 (25.7)		
PBSB, *n* (%): No	180 (47.4)	132 (48.0)	48 (45.7)	0.731	0.046
Yes	200 (52.6)	143 (52.0)	57 (54.3)		
YDBS, *n* (%): No	188 (49.5)	144 (52.4)	44 (41.9)	0.085	0.211
Yes	192 (50.5)	131 (47.6)	61 (58.1)		
DHBSO, *n* (%): No	187 (49.2)	143 (52.0)	44 (41.9)	0.086	0.203
Yes	193 (50.8)	132 (48.0)	61 (58.1)		
HTBS, *n* (%): No	208 (54.7)	143 (52.0)	65 (61.9)	0.085	0.201
Yes	172 (45.3)	132 (48.0)	40 (38.1)		
Age, years	79.00 [71.00, 86.00]	78.00 [71.00, 86.50]	79.00 [70.00, 84.00]	0.874	0.038
Height, m	1.58 [1.55, 1.65]	1.59 [1.55, 1.65]	1.58 [1.55, 1.60]	0.214	0.172
Weight, kg	55.00 [45.00, 60.00]	55.00 [45.00, 60.00]	55.00 [45.00, 60.00]	0.567	0.053
BMI, kg/m^2^	20.96 [18.12, 24.24]	20.87 [18.11, 24.24]	20.96 [18.49, 24.65]	0.851	0.013
Injury-to-surgery interval, h	144.00 [84.00, 216.00]	124.00 [80.00, 192.00]	168.00 [96.00, 240.00]	0.034	0.130
Immobilization duration, h	77.00 [60.00, 103.00]	75.00 [59.50, 101.00]	80.00 [64.00, 120.00]	0.054	0.206
PT, s	11.10 [10.70, 11.60]	11.10 [10.70, 11.50]	11.20 [10.60, 11.70]	0.913	0.221
INR	0.92 [0.89, 0.97]	0.92 [0.89, 0.96]	0.93 [0.88, 0.98]	0.743	0.267
APTT, s	25.10 [22.58, 29.50]	25.20 [22.55, 29.80]	24.40 [22.60, 27.50]	0.250	0.271
Fibrinogen, g/L	4.26 [3.41, 5.30]	4.08 [3.27, 5.12]	4.47 [4.11, 5.70]	<0.001	0.485
D-dimer, mg/L	7.05 [3.00, 15.18]	5.70 [2.45, 13.38]	8.94 [5.59, 29.14]	<0.001	0.635
CRP, mg/L	46.10 [20.28, 79.80]	38.60 [17.45, 76.00]	72.40 [26.80, 106.00]	<0.001	0.324
FBG, mmol/L	6.19 [5.65, 7.38]	6.08 [5.64, 7.04]	6.52 [5.68, 8.83]	0.009	0.407
TG, mmol/L	1.02 [0.81, 1.43]	1.01 [0.78, 1.30]	1.12 [0.86, 1.60]	0.007	0.288
TyG index	8.55 [8.29, 8.93]	8.54 [8.29, 8.85]	8.77 [8.28, 9.17]	0.001	0.463
TC, mmol/L	4.25 [3.56, 4.99]	4.24 [3.50, 5.00]	4.36 [3.85, 4.79]	0.183	0.139
HDL-C, mmol/L	1.23 [1.05, 1.46]	1.27 [1.07, 1.47]	1.10 [0.97, 1.44]	0.001	0.314
LDL-C, mmol/L	2.68 [2.06, 3.21]	2.57 [1.90, 3.17]	2.76 [2.24, 3.41]	0.047	0.013
WBC, ×10^9^/L	8.88 [7.29, 10.42]	9.16 [7.53, 10.40]	8.33 [6.84, 10.47]	0.150	0.033
Neutrophils, ×10^9^/L	6.95 [5.35, 8.22]	7.16 [5.60, 8.23]	6.12 [4.98, 8.05]	0.071	0.126
Lymphocytes, ×10^9^/L	1.21 [0.92, 1.55]	1.17 [0.90, 1.49]	1.26 [0.95, 1.64]	0.172	0.140
RBC, ×10^12^/L	3.72 [3.21, 4.18]	3.72 [3.28, 4.16]	3.70 [3.15, 4.22]	0.996	0.028
Hemoglobin, g/L	116.00 [98.00, 130.00]	117.00 [102.00, 130.00]	105.00 [90.00, 125.00]	0.001	0.348
Platelets, ×10^9^/L	222.00 [187.00, 270.00]	217.00 [176.50, 260.00]	254.00 [202.00, 352.00]	<0.001	0.625

Data are median (IQR) or *n* (%). Continuous variables were compared with the Mann–Whitney U test and categorical variables with Fisher’s exact test. SMD denotes standardized mean difference. TyG was recalculated from TG and FBG using consistent units. QDBS, Qi deficiency with blood stasis; QSBS, Qi stagnation with blood stasis; CCBS, cold coagulation with blood stasis; PBSB, phlegm-blood stasis binding; YDBS, Yin deficiency and blood stasis; DHBSO, dampness-heat and blood stasis obstruction; HTBS, heat-toxin and blood stasis.

### 3.2 Group-constrained partition and predictor panel

The 380 patients formed 243 distinct exact multivariable profiles, including 101 profiles represented by more than one patient. Group-constrained partitioning assigned 267 patients with 74 DVT events to the development set and 113 patients with 31 events to the held-out validation set. DVT incidence was similar between sets (27.7% vs. 27.4%), and no exact profile was shared across the partition.

Some covariate imbalance remained after the group-constrained split. The largest absolute standardized mean differences were observed for RBC (0.313), CRP (0.257), FBG (0.241), YDBS (0.226), and QSBS (0.226) (Figure 2; Supplementary Table S1).

**Figure 2:**
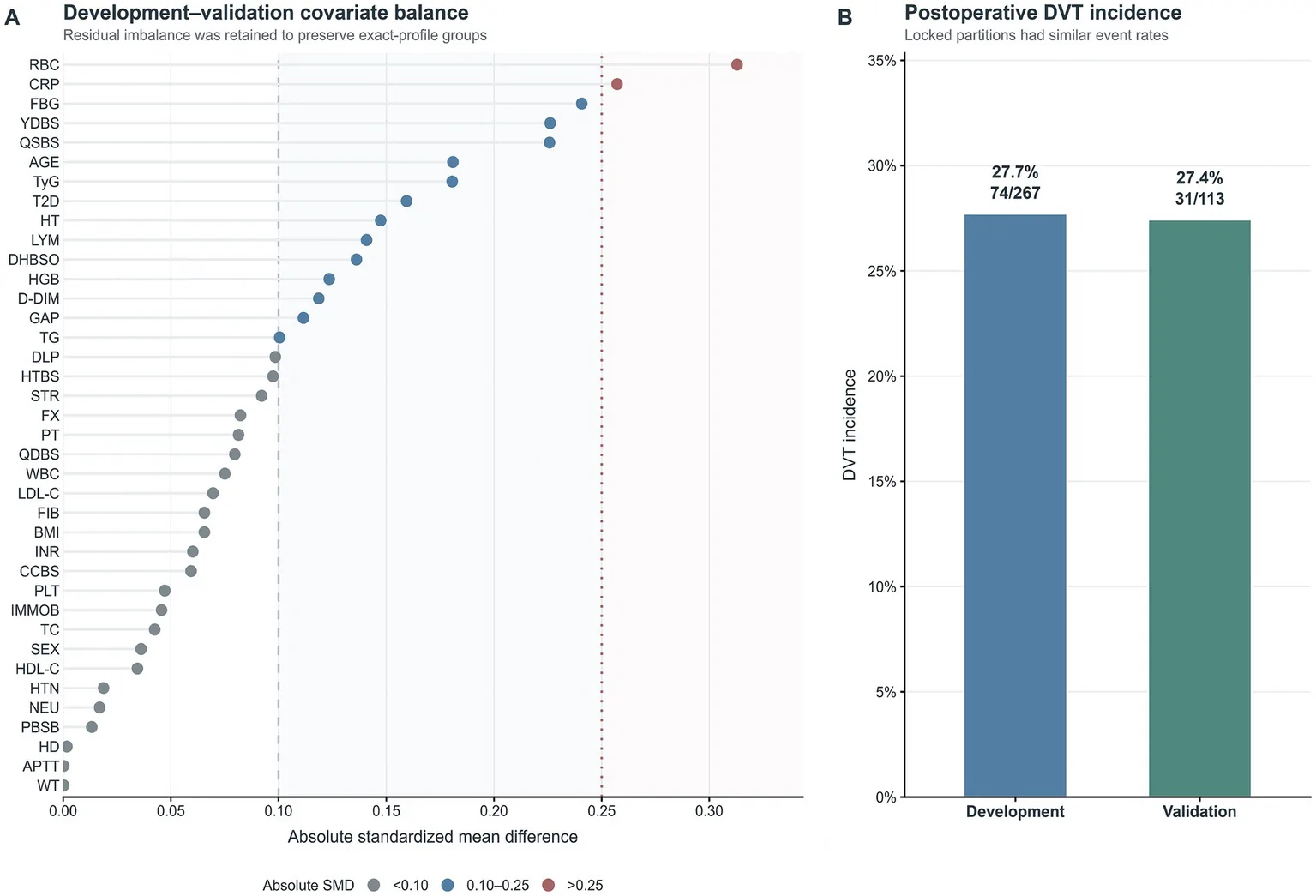
Baseline comparability between the development and held-out internal validation sets. **(A)** Absolute standardized mean differences (SMDs) for 38 baseline variables after profile-aware partitioning. Dashed and dotted lines indicate |SMD| = 0.10 and 0.25, respectively. Fifteen variables exceeded 0.10 and two exceeded 0.25, with the largest imbalances for RBC, CRP, FBG, YDBS, and QSBS. Exact-profile groups were kept intact during partitioning. **(B)** Postoperative DVT incidence was 27.7% (74/267) and 27.4% (31/113) in the development and validation sets, respectively. FX, fracture-type code; QDBS, Qi Deficiency with Blood Stasis; QSBS, Qi Stagnation with Blood Stasis; YDBS, Yin Deficiency and Blood Stasis.

The fixed eight-variable predictor panel comprised D-dimer, fibrinogen, platelet count, T2D, FBG, TyG, QSBS, and QDBS (Table 2).

**Table 2:** Fixed eight-variable predictor panel and SHAP importance of the RF index model.

Predictor	Category	Mean absolute SHAP	SHAP rank
D-dimer	Clinical/laboratory	0.0926	1
FBG	Clinical/laboratory	0.0700	2
Fibrinogen	Clinical/laboratory	0.0697	3
Platelet count	Clinical/laboratory	0.0445	4
T2D	Clinical/laboratory	0.0344	5
TyG index	Clinical/laboratory	0.0253	6
QSBS	TCM syndrome	0.0171	7
QDBS	TCM syndrome	0.0098	8

SHAP values were calculated for the RF index model in the profile-disjoint validation set (*n* = 113; 31 DVT events). The eight-variable predictor panel was fixed before held-out validation and used unchanged in the analysis. SHAP values quantify contributions to model predictions and should not be interpreted as causal effects.

### 3.3 Discrimination and internal stability

During group-aware development resampling, RF achieved the highest pooled OOF AUC among the nine algorithms at 0.774 (95% CI 0.710–0.837); development OOF AUCs for the other algorithms ranged from 0.608 for KNN to 0.770 for AdaBoost (Table 3). RF was therefore carried forward as the development-selected index model.

**Table 3:** Development and validation discrimination of the nine machine-learning algorithms.

Model	Development pooled group-aware OOF AUC (95% CI)	Profile-disjoint validation AUC (95% CI)
Logistic	0.732 (0.660–0.804)	0.839 (0.757–0.921)
SVM	0.755 (0.686–0.825)	0.873 (0.803–0.943)
KNN	0.608 (0.533–0.683)	0.622 (0.523–0.722)
NN	0.764 (0.697–0.831)	0.810 (0.717–0.903)
RF	0.774 (0.710–0.837)	0.859 (0.782–0.935)
GBM	0.745 (0.680–0.810)	0.868 (0.799–0.938)
AdaBoost	0.770 (0.709–0.831)	0.782 (0.691–0.872)
XGBoost	0.752 (0.691–0.813)	0.852 (0.780–0.924)
LightGBM	0.747 (0.685–0.809)	0.830 (0.751–0.910)

The same fixed eight-variable predictor panel was used for all algorithms. Development AUCs are pooled group-aware OOF estimates; validation AUCs are from the common profile-disjoint held-out cohort (*n* = 113; 31 DVT events). AUC confidence intervals were calculated by the DeLong method. RF was selected as the index model using development-stage OOF performance before held-out validation.

In the profile-disjoint validation set, AUCs ranged from 0.622 for KNN to 0.873 for SVM (Figure 3; Table 3). SVM (0.873), GBM (0.868), and RF (0.859; 95% CI 0.782–0.935) had the highest validation AUCs. Pairwise DeLong comparisons did not show significant differences between RF and SVM or GBM (*p* = 0.532 and *p* = 0.475, respectively; Supplementary Table S2).

**Figure 3:**
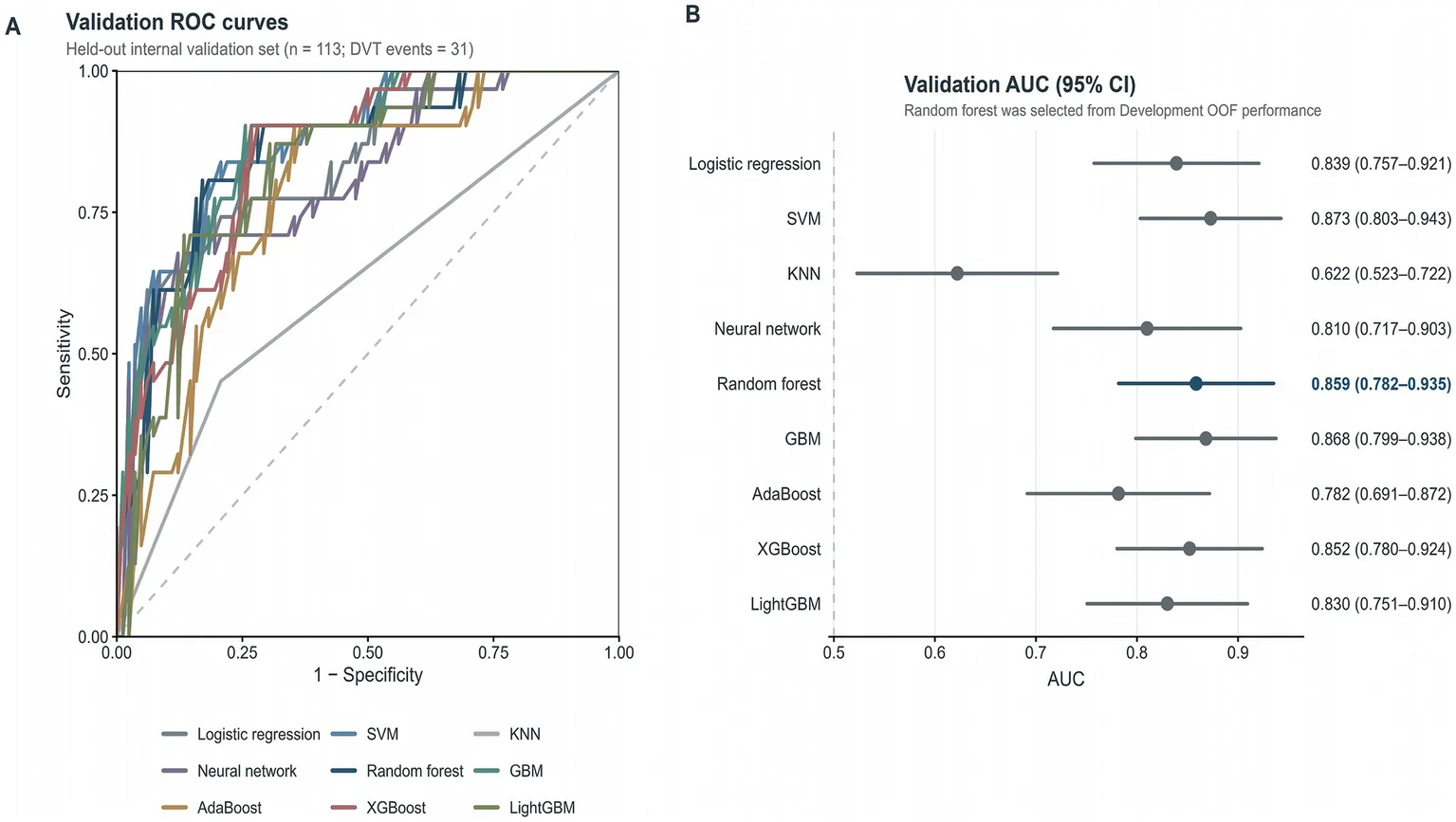
Discrimination of the nine machine-learning algorithms in the held-out internal validation set (*n* = 113; 31 postoperative DVT events). **(A)** Receiver operating characteristic (ROC) curves. **(B)** Area under the ROC curve (AUC) with 95% confidence intervals. Random forest was selected as the index model according to development-set out-of-fold performance before the held-out validation set was examined; validation-set AUC ranking was descriptive and was not used for model selection.

Across 30 repeated group-constrained development-validation partitions, RF had a mean held-out AUC of 0.792 (SD 0.054), a median of 0.795, and a 2.5th–97.5th percentile range of 0.701–0.887 (Supplementary Table S6).

In the fold-nested SMOTENC sensitivity analysis, RF validation AUC was 0.859, essentially unchanged from the primary no-SMOTE analysis (ΔAUC = 0.0006; paired DeLong *p* = 0.985) (Supplementary Table S5).

### 3.4 Incremental predictive value of TCM syndrome variables

Using the same RF algorithm and validation cohort, the six-variable clinical/laboratory model achieved an AUC of 0.855 and the eight-variable model including QSBS and QDBS achieved an AUC of 0.859. The absolute difference was small and not statistically significant (ΔAUC = +0.003; paired DeLong *p* = 0.746).

The Brier score decreased from 0.134 to 0.132 after adding QSBS and QDBS. Calibration slopes were 1.163 and 1.136, and calibration intercepts were −0.131 and −0.305, for the clinical/laboratory and integrated models, respectively. Secondary reclassification measures favored the integrated model: continuous NRI was 0.621 (95% CI 0.263–0.943) and IDI was 0.035 (95% CI 0.017–0.053) (Supplementary Table S4).

The AUC difference between the integrated and clinical/laboratory RF models was minimal and non-significant, whereas the secondary NRI and IDI estimates were positive.

### 3.5 Operating characteristics, calibration, and clinical utility

At the development-derived threshold of 0.298, RF identified 28 of 31 DVT events and correctly classified 59 of 82 non-DVT patients, yielding 28 true positives, 23 false positives, 59 true negatives, and 3 false negatives (Figure 4A). Sensitivity was 0.903, specificity 0.720, accuracy 0.770, PPV 0.549, NPV 0.952, and F1-score 0.683 (Table 4). RF PR-AUC was 0.708; precision-recall curves for all nine algorithms are shown in Supplementary Figure S1.

**Figure 4:**
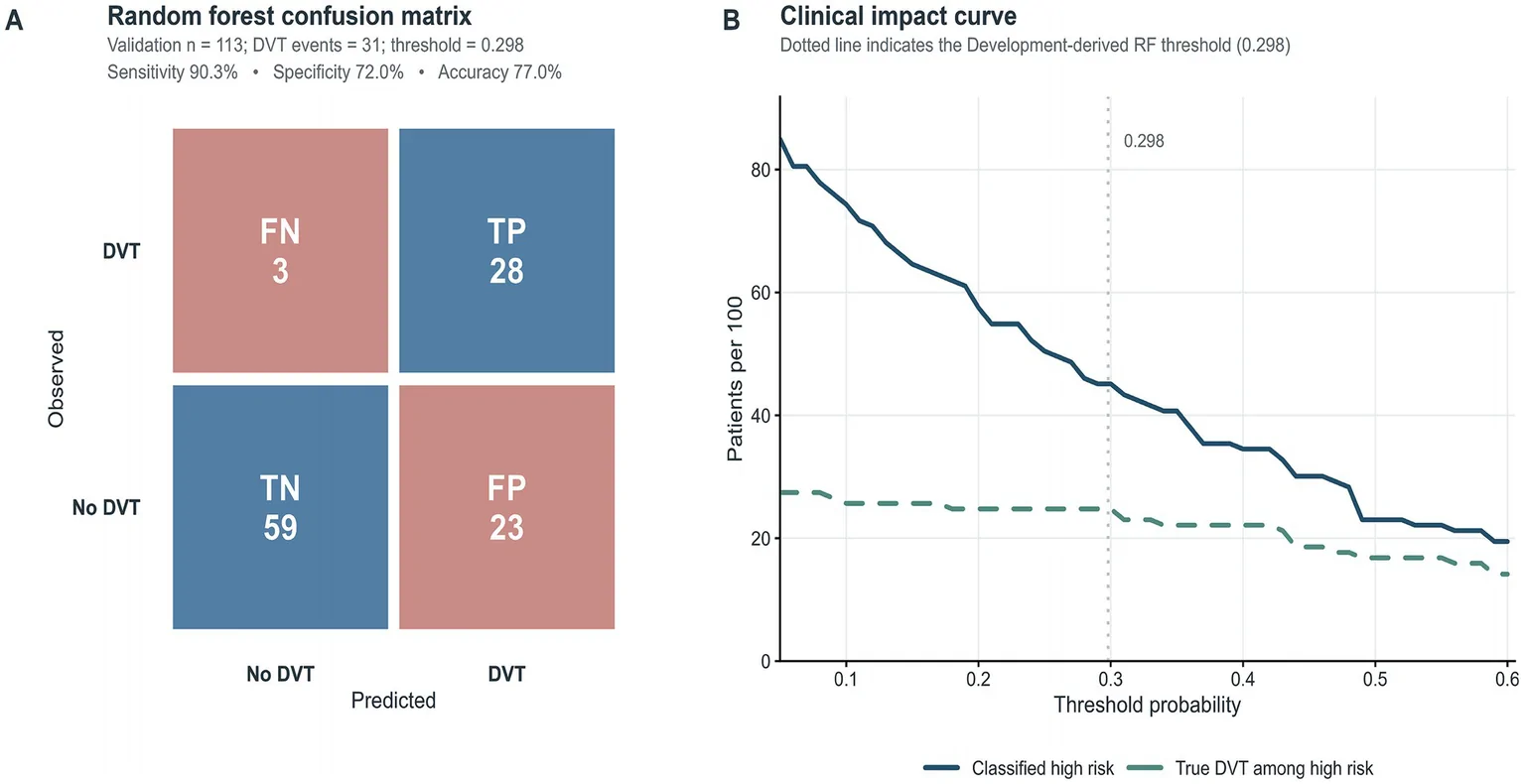
Random-forest classification performance and clinical-impact analysis in the held-out validation set. **(A)** Confusion matrix for the RF index model at the development-derived OOF Youden threshold of 0.298 in the validation cohort (*n* = 113; 31 DVT events): TP = 28, FP = 23, TN = 59, and FN = 3. **(B)** Clinical-impact analysis showing predicted high-risk patients and true-positive patients per 100 across threshold probabilities.

**Table 4:** Validation performance of the nine machine-learning models.

(A) Threshold-based classification performance								
Model	Threshold	TP/FP/TN/FN	Sensitivity	Specificity	Accuracy	PPV	NPV	F1
Logistic	0.386	19/7/75/12	0.613	0.915	0.832	0.731	0.862	0.667
SVM	0.433	16/4/78/15	0.516	0.951	0.832	0.800	0.839	0.627
KNN	0.047	14/17/65/17	0.452	0.793	0.699	0.452	0.793	0.452
NN	0.190	26/41/41/5	0.839	0.500	0.593	0.388	0.891	0.531
RF	0.298	28/23/59/3	0.903	0.720	0.770	0.549	0.952	0.683
GBM	0.286	28/25/57/3	0.903	0.695	0.752	0.528	0.950	0.667
AdaBoost	0.420	28/41/41/3	0.903	0.500	0.611	0.406	0.932	0.560
XGBoost	0.199	28/36/46/3	0.903	0.561	0.655	0.438	0.939	0.589
LightGBM	0.180	27/27/55/4	0.871	0.671	0.726	0.500	0.932	0.635
Thresholds were derived exclusively from development-set OOF predictions using Youden’s index and applied unchanged to the profile-disjoint validation set. Values are point estimates in *n* = 113 patients with 31 DVT events.								

(B) Probabilistic discrimination and calibration performance							
Model	PR-AUC	Calibration intercept	Calibration slope	O: E ratio	Brier	Scaled Brier	H–L *p-*value
Logistic	0.631	−0.432	0.696	0.915	0.133	0.333	0.327
SVM	0.664	−0.572	0.582	0.900	0.125	0.372	0.108
KNN	0.383	−0.768	0.042	1.000	0.301	−0.511	–
NN	0.611	−0.242	0.915	0.914	0.139	0.300	0.411
RF	0.708	−0.305	1.136	0.846	0.132	0.338	0.314
GBM	0.731	−0.161	1.297	0.859	0.129	0.352	0.423
AdaBoost	0.500	−0.661	3.115	0.607	0.203	−0.022	<0.001
XGBoost	0.699	−0.179	1.009	0.917	0.137	0.311	0.543
LightGBM	0.569	−0.310	0.588	0.924	0.152	0.235	<0.001

Values are point estimates in the profile-disjoint validation set (*n* = 113; 31 DVT events). For the RF index model, the calibration slope was 1.136 (95% CI 0.753–1.867); this percentile bootstrap CI was estimated from 5,000 patient-level nonparametric bootstrap resamples of the held-out validation cohort. PR-AUC, area under the precision–recall curve; O: E, observed-to-expected ratio; H–L, Hosmer–Lemeshow; –, not reliably estimable because KNN produced only two distinct predicted probability values in the validation set.

The RF validation Brier score was 0.132 and the scaled Brier score was 0.338. Calibration intercept was −0.305, the calibration slope was 1.136 (95% CI 0.753–1.867), and the O: E ratio was 0.846; the Hosmer–Lemeshow *p-*value was 0.314 (Figure 5A; Table 4).

**Figure 5:**
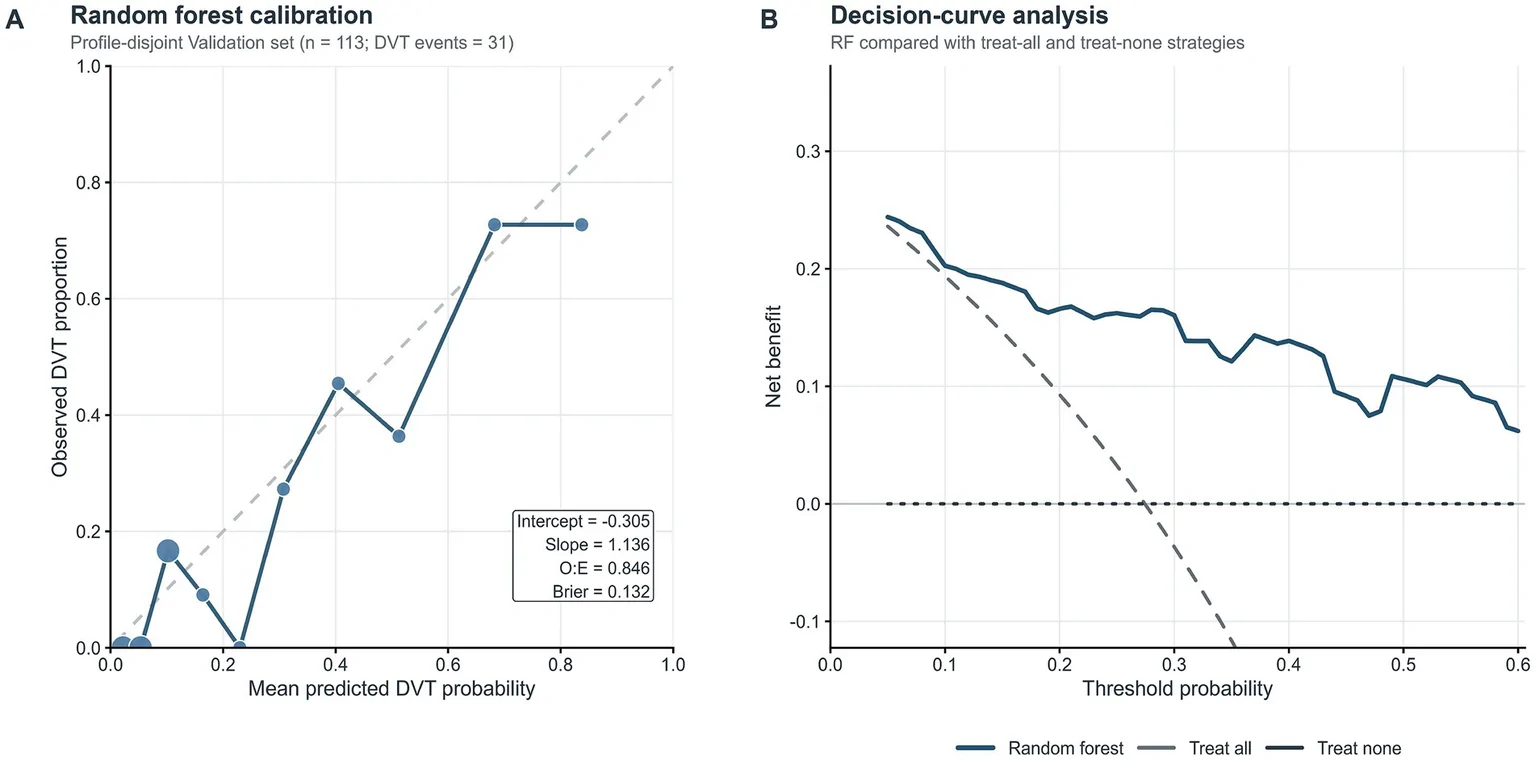
Calibration and decision-curve analysis of the RF index model. Analyses were performed in the profile-disjoint validation cohort (*n* = 113; 31 DVT events). **(A)** Calibration of predicted versus observed DVT probability; calibration intercept = −0.305, slope = 1.136 (95% CI 0.753–1.867), O: E = 0.846, and Brier score = 0.132. The dashed diagonal represents perfect calibration. **(B)** Decision-curve analysis comparing RF with treat-all and treat-none strategies across the displayed threshold range.

Decision-curve analysis showed positive net benefit for RF relative to the treat-none strategy and generally favorable net benefit relative to treat-all across the displayed threshold range (Figure 5B). The corresponding clinical-impact curve is shown in Figure 4B, and cross-model calibration and prediction-error estimates are presented in Figure 6. Descriptive RF subgroup validation by sex and age is provided in Supplementary Table S8.

**Figure 6:**
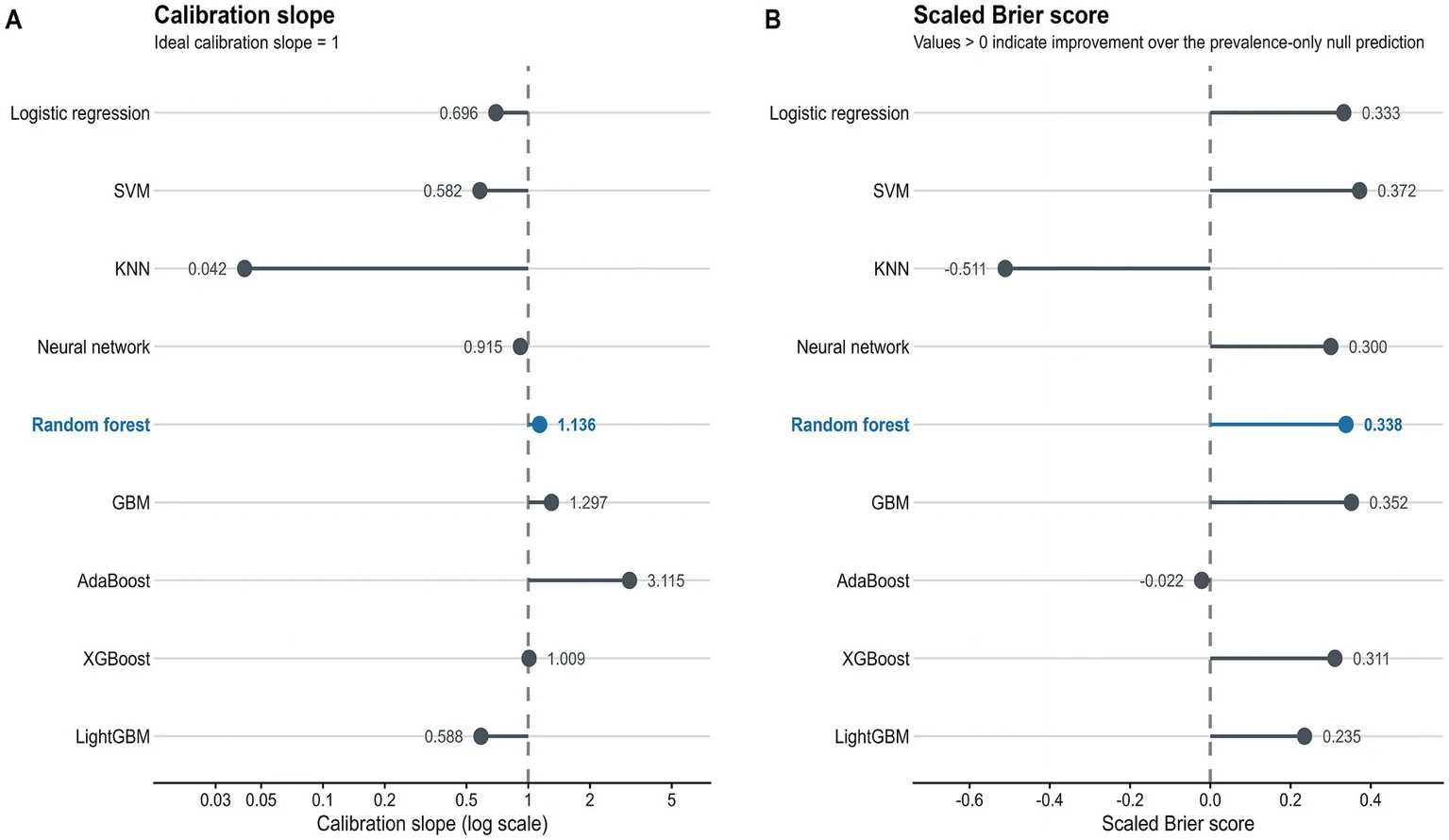
Cross-model calibration and prediction-error performance in the profile-disjoint held-out validation set (*n* = 113; 31 postoperative DVT events). **(A)** Calibration slopes for all nine algorithms displayed on a logarithmic scale; the dashed vertical line indicates the ideal calibration slope of 1. **(B)** Scaled Brier scores for the same nine algorithms; the dashed vertical line at 0 indicates no improvement over the prevalence-only null prediction, and larger values indicate greater improvement in overall probabilistic prediction error. Random forest is highlighted as the development-selected index model; the validation displays were descriptive and were not used for model reselection.

### 3.6 Model interpretability (SHAP)

SHAP analysis of the RF index model was performed in all 113 validation patients. D-dimer had the largest mean absolute SHAP value (0.0926), followed by FBG (0.0700), fibrinogen (0.0697), platelet count (0.0445), T2D (0.0344), TyG (0.0253), QSBS (0.0171), and QDBS (0.0098) (Figure 7; Table 2).

**Figure 7:**
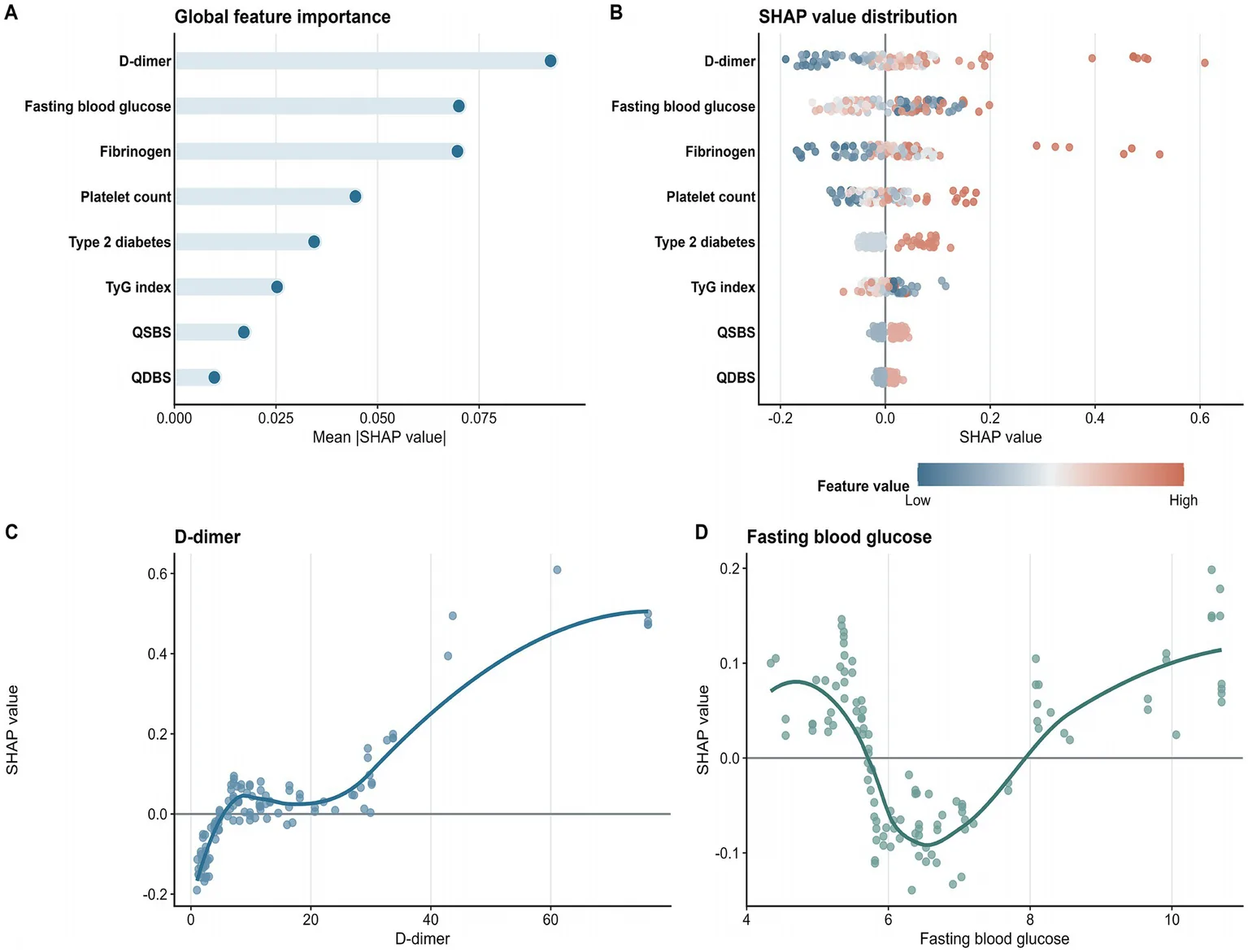
SHapley Additive exPlanations (SHAP) interpretation of the RF index model in the profile-disjoint held-out validation set (*n* = 113; 31 postoperative DVT events). **(A)** Global importance ranked by mean absolute SHAP value. **(B)** Distribution of patient-level SHAP contributions across the fixed eight predictors; the color gradient denotes low-to-high feature values within each predictor. **(C)** Dependence plot for D-dimer. **(D)** Dependence plot for fasting blood glucose. Positive SHAP values increase and negative SHAP values decrease the model-predicted DVT probability relative to the model baseline. The smooth curves in panels C and D are descriptive LOESS summaries of the saved feature-value/SHAP-value pairs. SHAP values describe contributions to model predictions and should not be interpreted as causal effects.

Clinical and laboratory variables occupied the six highest positions in the global RF importance ranking, whereas QSBS and QDBS ranked seventh and eighth, respectively.

### 3.7 Exploratory TyG interaction analyses

Among six exploratory interaction analyses, evidence of effect modification was observed for sex. The TyG-by-sex interaction had a likelihood-ratio *p-*value of 0.0056 and remained significant after FDR correction (FDR-adjusted *p* = 0.033). The OR per 1-SD higher TyG was 1.806 (95% CI 1.388–2.350) among women (86 events/268 patients) and 0.681 (95% CI 0.353–1.315) among men (19/112) (Figure 8; Supplementary Table S7).

**Figure 8:**
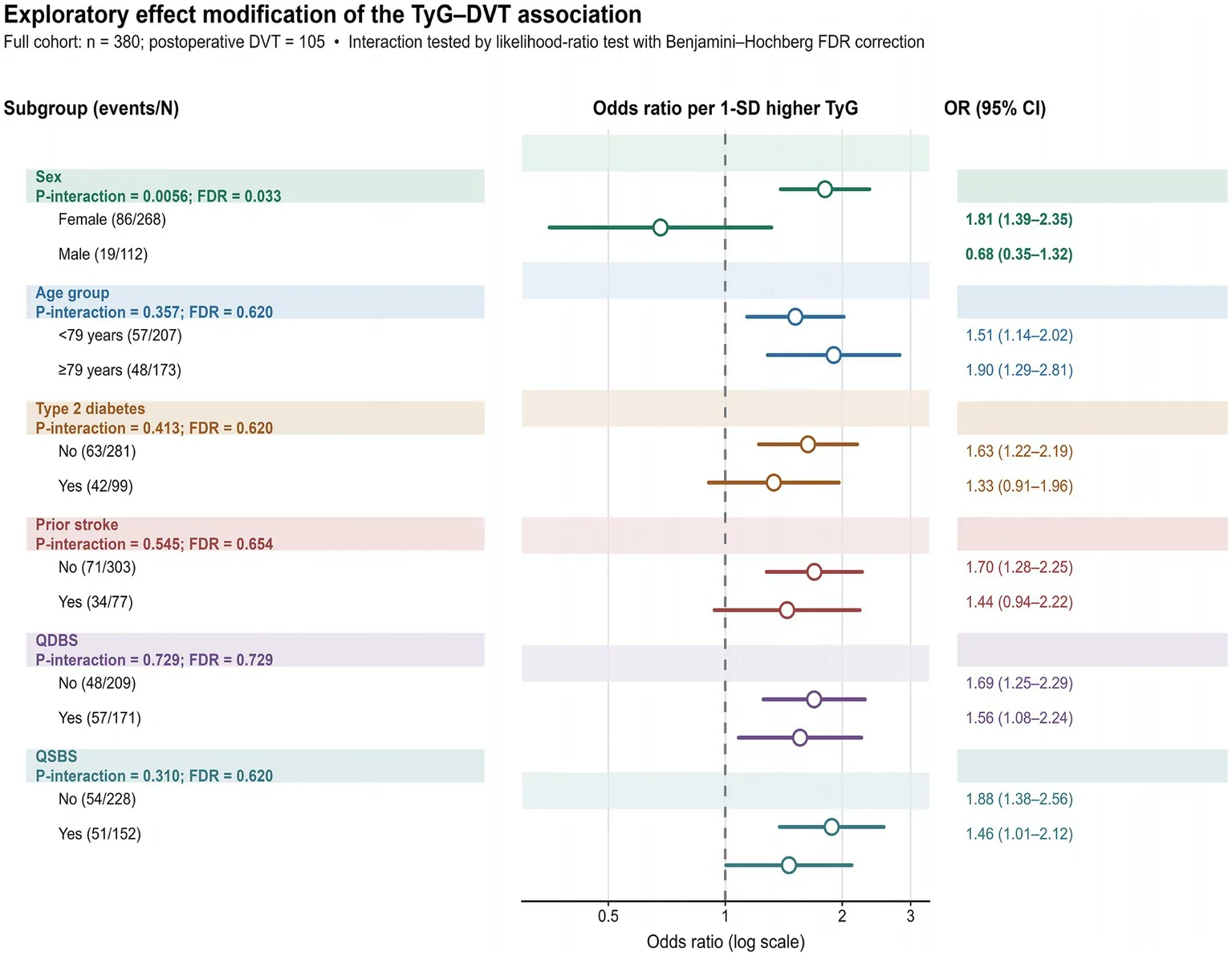
Exploratory interaction analyses for the corrected TyG index in the full cohort (*n* = 380; 105 postoperative DVT events). Points and horizontal lines show odds ratios (ORs) and 95% confidence intervals per 1-SD higher TyG within each subgroup. Interaction *p-*values were obtained from likelihood-ratio tests and adjusted across the six prespecified interaction tests using the Benjamini–Hochberg false discovery rate (FDR) procedure. The interaction models contained TyG, the subgroup variable, and their interaction term without additional covariate adjustment. The age-group cutoff was the development-set median (79 years). These analyses were exploratory and the sex-specific interaction should be interpreted as hypothesis-generating.

No interaction remained significant after FDR correction for age group, T2D, prior stroke, QDBS, or QSBS (all FDR-adjusted *p* ≥ 0.620).

### 3.8 Continuous TyG association and restricted cubic spline analysis

Higher TyG was associated with greater odds of postoperative DVT. In unadjusted analyses, the OR was 2.399 (95% CI 1.583–3.635; *p* < 0.001) per 1-unit increase and 1.625 (95% CI 1.290–2.047; *p* < 0.001) per 1-SD increase.

After adjustment for age, sex, T2D, D-dimer, fibrinogen, QDBS, and QSBS, the OR was 2.203 (95% CI 1.360–3.569; *p* = 0.001) per 1-unit increase and 1.550 (95% CI 1.186–2.026; *p* = 0.001) per 1-SD increase (Table 5).

**Table 5:** Association between corrected TyG and postoperative DVT.

Analysis	Contrast	OR (95% CI)	*p-*value
Unadjusted: per 1 TyG unit	Per 1-unit higher TyG	2.399 (1.583–3.635)	<0.001
Unadjusted: per 1 SD higher TyG	Per 1-SD higher TyG	1.625 (1.290–2.047)	<0.001
Unadjusted: high vs. low	TyG ≥ 8.609 vs. <8.609	2.150 (1.360–3.401)	0.001
Adjusted: per 1 TyG unit	Per 1-unit higher TyG	2.203 (1.360–3.569)	0.001
Adjusted: per 1 SD higher TyG	Per 1-SD higher TyG	1.550 (1.186–2.026)	0.001
Adjusted: high vs. low	TyG ≥ 8.609 vs. <8.609	1.710 (0.999–2.928)	0.050

Continuous TyG was the primary exposure. Adjusted models included age, sex, T2D, D-dimer, fibrinogen, QDBS, and QSBS. The secondary high-versus-low analysis used the development-set median of 8.609; this data-derived value should not be interpreted as a universal clinical threshold. APTT, activated partial thromboplastin time; AUC, area under the receiver operating characteristic curve; BMI, body mass index; CRP, C-reactive protein; DVT, deep vein thrombosis; FBG, fasting blood glucose; FDR, false discovery rate; GBM, gradient boosting machine; HDL-C, high-density lipoprotein cholesterol; IDI, integrated discrimination improvement; KNN, k-nearest neighbors; LDL-C, low-density lipoprotein cholesterol; LGBM, LightGBM; NPV, negative predictive value; NRI, net reclassification improvement; O: E, observed-to-expected ratio; OOF, out-of-fold; OR, odds ratio; PPV, positive predictive value; QDBS, Qi deficiency with blood stasis; QSBS, Qi stagnation with blood stasis; RCS, restricted cubic spline; RF, random forest; SHAP, SHapley Additive exPlanations; SMD, standardized mean difference; SVM, support vector machine; T2D, type 2 diabetes; TCM, Traditional Chinese Medicine; TG, triglycerides; TyG, triglyceride-glucose index; VTE, venous thromboembolism; XGB, XGBoost.

In the secondary dichotomized analysis using the development-set median of 8.609, TyG ≥ 8.609 was associated with higher odds of DVT in the unadjusted model (OR 2.150, 95% CI 1.360–3.401; *p* = 0.001). After multivariable adjustment, the estimate was attenuated (OR 1.710, 95% CI 0.999–2.928; *p* = 0.050).

The adjusted RCS analysis showed a significant overall association between TyG and postoperative DVT (*p* overall = 0.007), without significant evidence of nonlinearity (*p* nonlinear = 0.082) (Figure 9).

**Figure 9:**
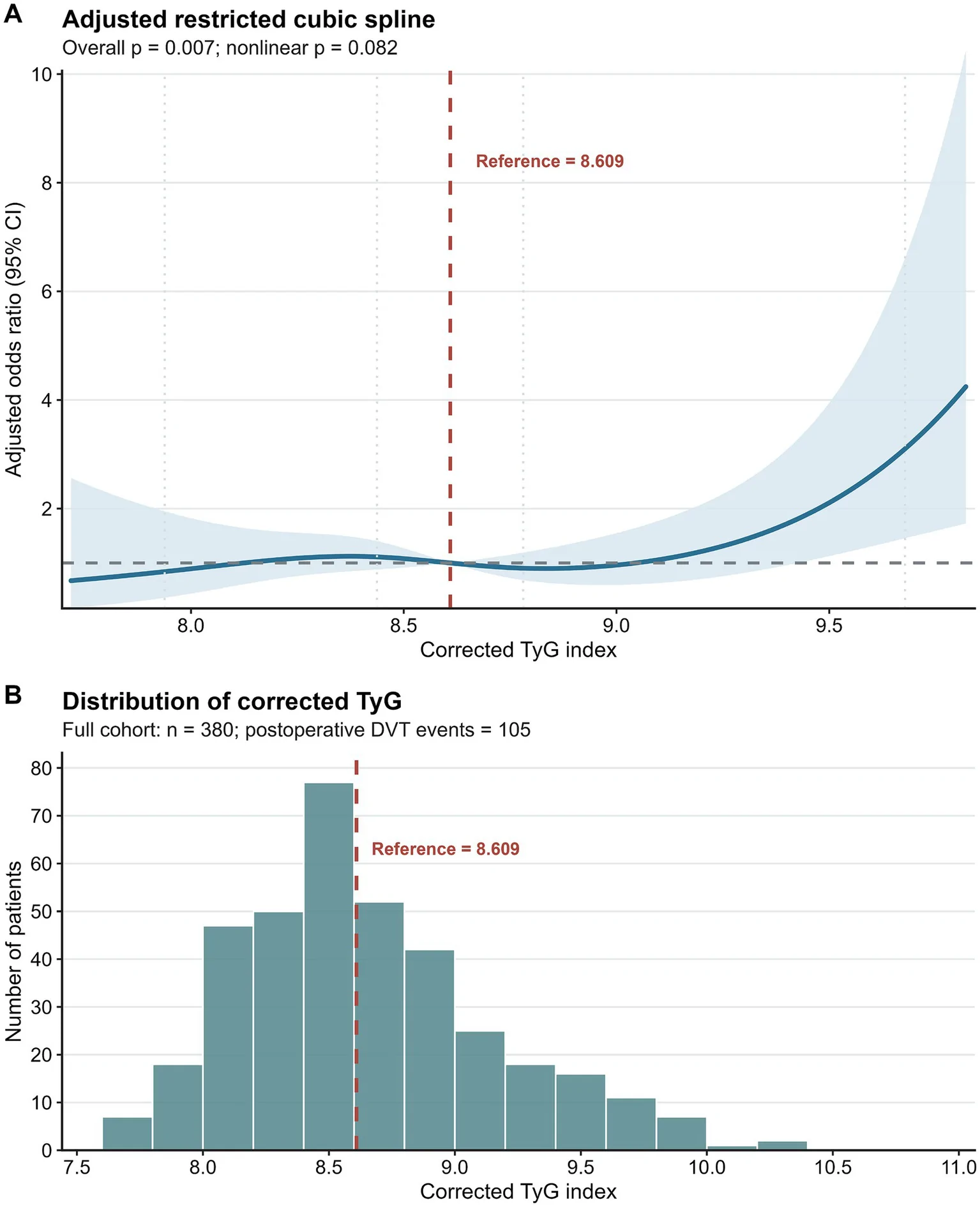
Corrected TyG association and distribution in the full cohort (*n* = 380; 105 postoperative DVT events). **(A)** Adjusted restricted cubic spline for corrected TyG and postoperative DVT, with knots at 7.938, 8.437, 8.781, and 9.678 and reference value 8.609 (*p* overall = 0.007; *p* nonlinear = 0.082). The model was adjusted for age, sex, T2D, D-dimer, fibrinogen, QDBS, and QSBS. **(B)** Distribution of corrected TyG values; the dashed vertical line indicates the reference value of 8.609.

## 4 Discussion

This study provides three principal findings. First, an RF model selected from development-set performance showed favorable discrimination in the profile-disjoint held-out cohort, although performance was more modest and variable across repeated group-constrained partitions. Second, QDBS and QSBS were more common among patients who developed DVT but did not provide a material increment in AUC when added to the clinical/laboratory RF model. Third, higher preoperative TyG remained associated with postoperative DVT after multivariable adjustment, with no statistically significant evidence of a nonlinear dose–response relationship.

The internal validation results highlight the uncertainty that can accompany model assessment in modest clinical datasets. The held-out RF AUC of 0.859 was higher than the mean AUC of 0.792 across 30 repeated group-constrained partitions, indicating sensitivity to the particular development-validation split. Reporting both estimates therefore provides a more representative account of internal performance than reliance on a single split. Although SVM and GBM achieved numerically higher AUCs in the held-out cohort, RF remained the primary model because model selection had been completed using development-set OOF performance before validation. This pattern is consistent with broader concerns that apparent gains from flexible ML algorithms can depend on sample size, validation design, and calibration rather than representing a stable advantage over simpler approaches (Rajkomar et al., 2019; Christodoulou et al., 2019; Wynants et al., 2017; Steyerberg et al., 2001; Collins et al., 2024).

Discrimination should be interpreted together with calibration and threshold-dependent performance. In the held-out cohort, the RF calibration slope was 1.136 (95% CI 0.753–1.867) and the O: E ratio was 0.846, consistent with overall overprediction of DVT risk. At the development-derived threshold of 0.298, PPV was 0.549, whereas NPV was 0.952; these predictive values are specific to the event prevalence and case-mix of this cohort. Decision-curve and clinical-impact analyses suggested potential net benefit over part of the evaluated threshold range, but these estimates remain internal and sample-dependent. External validation and, where necessary, recalibration are therefore prerequisites for clinical implementation.

The RF importance profile was dominated by conventional clinical and laboratory information. D-dimer and fibrinogen, which reflect activation of coagulation and fibrinolytic pathways and are repeatedly associated with DVT in hip-fracture populations, ranked among the strongest contributors (Wang et al., 2022; Song et al., 2016; Fu et al., 2020; Dou et al., 2022; Wolberg, 2023). FBG, T2D, and TyG indicate that metabolic status also contributed to risk discrimination, while platelet count added complementary hematologic information. These findings are biologically plausible but should not be interpreted causally because SHAP quantifies contributions to model predictions rather than etiologic effects. In addition, because TyG is mathematically derived in part from FBG, simultaneous inclusion of FBG and TyG may distribute importance across correlated metabolic features; individual SHAP ranks should therefore be interpreted cautiously.

The TCM findings illustrate an important distinction between association and incremental prediction. QDBS and QSBS were more prevalent in the DVT group, yet both ranked below the six clinical/laboratory predictors by mean absolute SHAP value, and their addition changed validation AUC only from 0.855 to 0.859. The favorable NRI and IDI estimates suggest that the integrated model altered individual risk ranking, but these were secondary measures and did not coincide with a statistically significant improvement in AUC. The present data therefore do not support a claim that the retained TCM syndrome variables materially improve discrimination. Whether more granular or more reproducibly measured TCM phenotypes add independent predictive information remains an open question that should be tested prospectively.

Higher TyG remained associated with postoperative DVT after adjustment for the multivariable adjustment set, with an OR of 1.550 per 1-SD increase. The spline analysis supported an overall association but did not provide statistically significant evidence for nonlinearity, arguing against interpretation of the development-set median of 8.609 as a biological or clinical threshold. This finding is directionally consistent with the positive association reported after total knee arthroplasty, but contrasts with the absence of an adjusted association for TyG alone in a large UK Biobank analysis of incident venous thromboembolism (Wu et al., 2025; Wang et al., 2026). Such heterogeneity may reflect differences in population, perioperative context, outcome ascertainment, and covariate structure. The TyG-by-sex interaction remained significant after FDR correction, but the limited number of male events and the exploratory, minimally adjusted interaction models make this result hypothesis-generating rather than a basis for sex-specific risk stratification.

### 4.1 Limitations

Several limitations should be considered. First, this was a retrospective single-center study with internal validation only; variation in AUC across repeated group-constrained partitions indicates that performance estimates remain sensitive to data partitioning and require external validation. Second, feature screening was performed once in the development set rather than nested within each resampling fold. Although the held-out validation set remained untouched, the selected predictor panel may be sample-dependent and development OOF estimates may retain some selection optimism. Third, group-constrained partitioning was used as a leakage-control safeguard and necessarily left some residual covariate imbalance between development and validation sets. Fourth, detailed pharmacologic thromboprophylaxis, anesthesia type, and several other perioperative variables were unavailable, leaving potential residual confounding and unmeasured predictive information. Fifth, the modeled outcome was inpatient lower-extremity DVT detected by routine ultrasonography; pulmonary embolism, proximal versus distal DVT, and symptomatic versus screening-detected events were not available as separate outcomes, limiting generalizability to other ascertainment strategies. Sixth, 48 patients were excluded because key baseline variables were missing, and the retained data do not support a detailed comparison with excluded patients. Source-record re-verification was targeted rather than a complete re-abstraction of all analytical variables: patient identity was re-verified for all analytical records using permanent hospital identifiers, whereas detailed source review focused on repeated-profile records and variables specifically flagged during data-quality review, including selected demographic and laboratory variables, DVT outcome, and injury-to-surgery interval. Seventh, only consensus TCM syndrome labels were retained, precluding formal assessment of inter-rater reliability. Finally, the exploratory interaction analyses were underpowered in some strata, and the mathematical relationship between FBG and TyG may affect attribution of importance among correlated metabolic predictors. Prospective multicenter studies are needed to evaluate transportability, recalibration, predictor stability, and reproducibility of the model, TyG association, and TCM findings.

## 5 Conclusion

In this single-center cohort, a development-selected RF model showed favorable discrimination in one profile-disjoint held-out validation set, but performance was more modest across repeated group-constrained partitions. Adding QDBS and QSBS did not materially improve AUC beyond clinical and laboratory predictors. Higher TyG remained associated with postoperative DVT after multivariable adjustment, without significant evidence of a nonlinear threshold effect. These findings support further evaluation of preoperative DVT risk stratification after hip arthroplasty for femoral neck fracture, but external validation and assessment of recalibration are required before clinical use.

## Data Availability

A de-identified patient-level analytical dataset supporting the reported analyses is supplied as Data Sheet 1 with the submission materials. Direct patient identifiers are not included in the analytical dataset and are not shared. The analysis script, model specifications, session information, and reproducibility materials supporting the reported tables and figures are supplied with the submission materials. Additional access to de-identified study data may be considered upon scientifically reasonable request to the corresponding authors, subject to institutional and ethical requirements.
