# Supplementary material for: Development and internal validation of machine-learning models for postoperative deep vein thrombosis after hip arthroplasty for femoral neck fracture: evaluation of Traditional Chinese Medicine syndrome features
Source: Front Artif Intell. 2026 Oct 29;9:1798230. doi: 10.3389/frai.2026.1798230 (PMC13755261; doi:10.3389/frai.2026.1798230)
Supplement: Supplementary file 1 [file Data_Sheet_3.PDF]

TRIPOD+AI 2024 Reporting Checklist — Final Current-Manuscript Mapping

Study: Development and Internal Validation of Machine-Learning Models for Postoperative Deep Vein Thrombosis After Hip Arthroplasty for Femoral Neck Fracture: Evaluation of Traditional Chinese Medicine Syndrome Features

Based on the TRIPOD+AI 27-item checklist (version 11 January 2024; Collins et al., BMJ 2024;385:e078378).

Purpose: submission mapping and final reporting audit. Page numbers refer to the current revised manuscript rendered on 17 August 2026; "Suppl." refers to the current figure-aligned Supplementary Materials. Items marked Partial are transparently reported limitations or implementation-stage details that are not fully applicable to this research-stage, single-centre development/internal-evaluation study.

Status: Complete = adequately mapped; Partial = present but not fully explicit; N/A = not applicable to this single-centre development/internal-evaluation study.

Section	Item	TRIPOD+AI requirement (paraphrased)	Status	Reported on	Current mapping / action
Title/Abstract	1	Identify prediction-model development/evaluation, target population, and predicted outcome.	**Complete**	p.1	Title names development/internal validation, population, procedure, and DVT outcome.
Title/Abstract	2	Report the abstract according to the TRIPOD+AI abstract guidance.	**Complete**	pp.1–2	Structured abstract reports design, cohort, split, predictors, selection, performance, calibration, TyG and conclusion.
Introduction	3a	Explain clinical context and rationale, including relevant existing models.	**Complete**	p.2	Orthopaedic DVT context, limitations of existing approaches, ML rationale, TCM and TyG background.
Introduction	3b	Describe target population, intended purpose in the care pathway, and intended users.	**Partial**	pp.2–3	Target population and risk-stratification purpose are clear; intended end users are not explicitly named.
Introduction	3c	Describe known health inequalities between sociodemographic groups.	**Partial**	pp.7–8; Suppl. p.7	Sex/age subgroup analyses are reported, but the Introduction does not explicitly discuss known health inequalities.
Introduction	4	State study objectives and whether model development and/or evaluation are performed.	**Complete**	p.3	Three explicit objectives; development and held-out internal evaluation are stated.
Methods — Data	5a	Describe data sources for development/evaluation, rationale, and representativeness.	**Complete**	p.3; Suppl. pp.1–2	Single-centre retrospective routine-care cohort; same source cohort partitioned profile-aware.
Methods — Data	5b	Give participant accrual dates and follow-up period when applicable.	**Complete**	p.3	October 2023 to June 2025; inpatient postoperative outcome ascertainment.
Methods — Participants	6a	Describe setting, number and location of centres.	**Complete**	p.3	Single-centre hospital cohort and institution are described.
Methods — Participants	6b	Report eligibility criteria.	**Complete**	p.3	Eligibility and exclusion criteria, including preoperative VTE, prior anticoagulant use and missing baseline variables.
Methods — Participants	6c	Describe relevant treatments received and how they were handled analytically.	**Partial**	p.3; p.10	All patients underwent hip arthroplasty; subtype not available. Postoperative pharmacologic thromboprophylaxis was not recorded, limiting treatment adjustment.
Methods — Data preparation	7	Describe preprocessing and data-quality checks, including demographic-group consistency where relevant.	**Complete**	pp.4–5; Suppl. pp.1–2, 8	Corrected TyG/BMI, identity/profile audit, missingness, preprocessing, group-constrained partition/resampling.
Methods — Outcome	8a	Define outcome, time horizon, assessment method/timing, rationale, and demographic-group consistency.	**Complete**	p.4	Postoperative lower-extremity DVT during hospitalization by routine duplex ultrasound; PE not modelled.
Methods — Outcome	8b	If outcome assessment is subjective, describe assessor qualifications/demographics.	**Partial**	p.4	Outcome modality is stated, but qualifications/demographics of ultrasound assessors are not reported.
Methods — Outcome	8c	Report any blinding of outcome assessment.	**Partial**	p.4	No explicit statement on blinding of duplex outcome assessors; do not infer blinding.
Methods — Predictors	9a	Explain initial predictor choice and any preselection before model building.	**Complete**	pp.4–5; Suppl. p.2	Prespecified TyG/QDBS/QSBS plus development-set screening under the event constraint.
Methods — Predictors	9b	Define predictors and when/how they were measured; report any predictor-assessment blinding.	**Complete**	pp.3–4	Demographics/comorbidities/labs and preoperative timing are described; TyG formula specified.
Methods — Predictors	9c	For subjectively assessed predictors, report assessor qualifications/demographics.	**Complete**	p.4	Two senior TCM physicians (>10 years' experience) and consensus process are described.
Methods — Sample size	10	Explain how study size was obtained and justify adequacy, including any formal calculation.	**Partial**	pp.3, 5–6; Table 4 p.26	Consecutive eligible cohort and 74 development events/8 predictors (9.25 events per parameter) are reported; no formal a priori sample-size calculation is reported.
Methods — Missing data	11	Describe handling of missing data and reasons for omitted records.	**Complete**	pp.3–4; Suppl. p.8	48 excluded before final cohort; final N=380 has no missing values; planned median imputation was not required.
Methods — Analysis	12a	Explain use/partition of data for development and performance evaluation.	**Complete**	pp.4–5; Suppl. pp.1–2	Profile-aware 70/30 development/validation partition with zero profile overlap.
Methods —	12b	Describe predictor handling, transformations, rescaling and	**Complete**	pp.4–5	TyG derivation, preprocessing, scaling/standardisation, KNN PCA and final fixed predictor

Section	Item	TRIPOD+AI requirement (paraphrased)	Status	Reported on	Current mapping / action
Analysis		standardisation.			panel are described.
Methods — Analysis	12c	Describe model types, rationale, model-building steps, tuning and internal validation.	**Complete**	pp.4–5; Suppl. pp.2, 9	Nine algorithms, repeated group-aware 10-fold CV ×5, RF development-OOF selection, hyperparameters in S10.
Methods — Analysis	12d	Describe cluster heterogeneity handling if relevant.	**N/A**	—	Single-centre study; no hospital/country cluster heterogeneity analysis was applicable.
Methods — Analysis	12e	Specify performance measures/plots and model comparisons.	**Complete**	p.5; pp.7–8; Figs.3–6 pp.19–25	AUC/CI, PR-AUC, threshold metrics, calibration, DCA, clinical-impact and DeLong comparisons.
Methods — Analysis	12f	Describe any model updating after evaluation.	**N/A**	—	No model updating/recalibration was performed after held-out evaluation.
Methods — Analysis	12g	For model evaluation, state how model predictions were generated.	**Partial**	p.5; reproducibility package	Prediction workflow is implicit in the saved fitted-model/prediction artifacts; manuscript does not give a standalone formula/API description for each algorithm.
Methods — Class imbalance	13	If imbalance methods are used, explain rationale/method and recalibration if needed.	**Complete**	pp.4–5; p.7; Suppl. p.5	Primary no-balancing analysis; fold-nested SMOTENC only as a sensitivity analysis.
Methods — Fairness	14	Describe fairness approaches and rationale.	**Partial**	p.5; pp.7–8; Suppl. p.7	Sex/age subgroup performance and exploratory TyG effect modification are reported, but no formal fairness intervention was prespecified.
Methods — Model output	15	Specify prediction output and rationale for classification thresholds.	**Complete**	pp.4–5; p.7	Probabilities plus development-OOF Youden threshold; threshold applied unchanged to validation.
Methods — Training vs evaluation	16	Identify differences between development and evaluation data/settings/outcomes/predictors.	**Complete**	p.6; Fig.2 p.18; Suppl. pp.2–3	Same source setting/criteria/outcome/predictor definitions; residual covariate imbalance reported with SMDs.
Methods — Ethics	17	Name ethics board and report consent or waiver.	**Complete**	p.11	Gangcheng Rehabilitation Hospital Ethics Committee, approval 2025-SR-779; consent waived.
Open science	18a	Report funding source and funder role.	**Complete**	p.11	Funding statement present.
Open science	18b	Declare conflicts of interest/financial disclosures.	**Complete**	p.11	Conflict-of-interest statement present.
Open science	18c	State where the protocol is available, or state that none was prepared.	**Complete**	p.11	A formal study protocol was prepared before study conduct and is available from the corresponding author upon reasonable request.
Open science	18d	Give registration details, or state that the study was not registered.	**Complete**	p.11	The study was not registered.
Open science	18e	Describe data availability.	**Complete**	p.11	De-identified patient-level Data Sheet 1 supplied; direct identifiers excluded; further access subject to institutional/ethical constraints.
Open science	18f	Describe analytical-code availability.	**Complete**	p.11; reproducibility package	Current final reproducibility package includes analysis/verification code and locked source artifacts.
Patient/public involvement	19	Describe patient/public involvement or state that there was none.	**Complete**	p.11	Patients and the public were not involved in the design, conduct, reporting, interpretation, or dissemination of this study.
Results — Participants	20a	Report participant flow and outcome counts; diagram if useful.	**Complete**	p.6; Fig.1 p.15	481 assessed, 101 excluded with reasons, 380 analysed, 105 DVT.
Results — Participants	20b	Report participant characteristics, key dates/predictors/treatments/events/missing data and demographic differences.	**Complete**	p.6; Table 1 pp.16–17; Suppl. pp.2–3	Full cohort and outcome-stratified characteristics reported; missingness audit in supplement.
Results — Participants	20c	For evaluation, compare important predictor/outcome distributions with development data.	**Complete**	p.6; Fig.2 p.18; Suppl. pp.2–3	Development-validation SMDs and DVT incidence reported.
Results — Model development	21	Report participant and event counts for each analysis stage.	**Complete**	pp.1, 6–8; Suppl. pp.1–2	Development 267/74; validation 113/31; subgroup/event counts reported where relevant.
Results — Model specification	22	Provide enough final-model detail to permit prediction/evaluation in new individuals, including access restrictions.	**Complete**	Suppl. p.9; reproducibility package	Final predictor panel, RF engine/hyperparameters, serialized RF object and verification materials supplied.
Results — Performance	23a	Report performance with confidence intervals, including key subgroups where relevant.	**Complete**	pp.6–8; Tables 2–3 pp.20, 22–23; Suppl. pp.4, 7	Validation AUCs with 95% CIs; RF operating/calibration metrics; sex/age subgroup results.
Results — Performance	23b	Report heterogeneity across clusters if examined.	**N/A**	—	No multi-centre/cluster heterogeneity analysis.
Results — Updating	24	Report results of any model updating.	**N/A**	—	No post-evaluation updating was undertaken.

Section	Item	TRIPOD+AI requirement (paraphrased)	Status	Reported on	Current mapping / action
Discussion	25	Interpret main findings, including fairness considerations, relative to objectives and prior evidence.	**Complete**	pp.8–10	Moderate interpretation, model-selection context, sex interaction caution, TCM/TyG interpretation.
Discussion	26	Discuss limitations and implications for bias, uncertainty and generalisability.	**Complete**	pp.9–10	Single-centre design, sample size, split variability, unavailable treatment/anesthesia data, outcome limitations, excluded missing records, TCM reliability limitations.
Discussion — Usability	27a	Explain how poor-quality/unavailable input data should be handled during implementation.	**Partial**	pp.9–10	Model is explicitly not ready for clinical implementation; no operational missing-input handling rule is specified.
Discussion — Usability	27b	State whether user interaction/expertise would be required in handling inputs or using the model.	**Partial**	pp.9–10	No deployment interface or intended user workflow is specified because clinical implementation is not proposed.
Discussion — Next steps	27c	Describe future research for applicability/generalizability.	**Complete**	pp.9–10	External multicentre validation, recalibration/transportability and prospective evaluation are recommended.

Final integrity note: this mapping does not infer unavailable information. Items marked Partial or N/A reflect the scope of the current research-stage study and should not be converted to affirmative claims without supporting source information.
