# Supplementary material for: Development and internal validation of machine-learning models for postoperative deep vein thrombosis after hip arthroplasty for femoral neck fracture: evaluation of Traditional Chinese Medicine syndrome features
Source: Front Artif Intell. 2026 Oct 29;9:1798230. doi: 10.3389/frai.2026.1798230 (PMC13755261; doi:10.3389/frai.2026.1798230)
Supplement: Supplementary file 2 [file Data_Sheet_1.docx]

**Supplementary Materials**

Development and Internal Validation of Machine-Learning Models for Postoperative Deep Vein Thrombosis After Hip Arthroplasty for Femoral Neck Fracture: Evaluation of Traditional Chinese Medicine Syndrome Features

**Supplementary material index**

Supplementary Methods. Profile-aware partitioning, leakage audit, and additional analysis details.

Supplementary Table S1. Development-validation baseline balance after profile-aware partitioning.

Supplementary Table S2. Pairwise DeLong comparisons among the nine validation AUCs (36 comparisons).

Supplementary Table S3A. Pairwise phi correlations among the seven TCM syndrome indicators.

Supplementary Table S3B. Variance-inflation factors for the seven TCM syndrome indicators.

Supplementary Table S4. Incremental predictive value of QDBS and QSBS.

Supplementary Table S5. Primary no-SMOTE RF analysis versus fold-nested SMOTENC sensitivity analysis.

Supplementary Table S6A. Thirty repeated profile-aware development-validation partitions.

Supplementary Table S6B. Summary of the 30 repeated profile-aware RF AUCs.

Supplementary Table S7. Exploratory TyG interaction analyses with likelihood-ratio testing and FDR correction.

Supplementary Table S8A-B. Descriptive RF subgroup validation by sex and age.

Supplementary Table S9. Missingness and imputation audit.

Supplementary Table S10. Model engines, selected hyperparameters, and reproducibility specifications.

Supplementary Figure S1. Precision-recall curves for all nine models in the profile-disjoint validation set.

**Supplementary Methods**

**Patient-identity verification**

Each of the 380 analytical records was confirmed to represent a unique patient during cohort assembly using permanent hospital patient identifiers. Direct identifiers were removed before analytical processing. Repeated exact clinical profiles therefore represented different patients rather than duplicate patient records.

**Exact-profile grouping and leakage control**

Exact clinical profiles were defined by simultaneous agreement across the following 23 prespecified variables: sex, age, height, weight, hypertension, type 2 diabetes, heart disease, prior stroke, dyslipidemia, the recorded fracture-type field, injury-to-surgery interval, immobilization duration, PT, INR, APTT, fibrinogen, D-dimer, CRP, FBG, TG, RBC, hemoglobin, and platelet count. The final cohort contained 243 exact profiles, including 101 profile groups represented by more than one distinct patient. The profile-size distribution comprised 142 singletons, 72 pairs, 23 triplets, 5 quadruplets, and 1 quintuplet. All members of an exact profile were kept within the same development-validation partition and within the same development-set resampling fold. The final development set comprised 267 patients with 74 DVT events, and the held-out validation set comprised 113 patients with 31 events. Exact-profile overlap between development and validation was zero, and all 50 development resampling folds had zero exact-profile overlap between analysis and assessment subsets.

**Model development and sensitivity analyses**

The eight-predictor panel was fixed as D-dimer, fibrinogen, platelet count, type 2 diabetes, fasting blood glucose, TyG, QSBS, and QDBS. Nine algorithms were evaluated with repeated 10-fold cross-validation (five repeats) under the exact-profile grouping constraint. Random forest was selected as the index model using development-set out-of-fold AUC before held-out validation. The primary analysis used the observed class distribution; fold-nested SMOTENC was used only as a sensitivity analysis. Classification thresholds were derived from pooled development-set out-of-fold predictions and applied unchanged to held-out validation.

**Additional data elements not available for valid analysis**

Patient-level details of postoperative pharmacologic thromboprophylaxis, anesthesia type, and operative duration were not recorded in the retained analytical dataset. Arthroplasty subtype was not available as a validated analytical variable. The exact postoperative day of venous ultrasonography could not be reconstructed reliably for every patient, and pulmonary embolism and detailed thrombus characteristics were not included in the modeled outcome. Separate physician-level TCM ratings were not retained, precluding reconstruction of inter-rater kappa. These unavailable elements were not inferred, imputed, or coded as absent.

**Supplementary Table S1. Development-validation baseline balance after profile-aware partitioning.**

| **Variable** | **Development** | **Validation** | **Absolute SMD** |
| --- | --- | --- | --- |
| n | 267 | 113 |  |
| Sex, n (%): Female | 187 (70.0) | 81 (71.7) | 0.036 |
| Male | 80 (30.0) | 32 (28.3) |  |
| Hypertension, n (%): No | 123 (46.1) | 51 (45.1) | 0.019 |
| Yes | 144 (53.9) | 62 (54.9) |  |
| Type 2 diabetes, n (%): No | 192 (71.9) | 89 (78.8) | 0.159 |
| Yes | 75 (28.1) | 24 (21.2) |  |
| Prior stroke, n (%): No | 210 (78.7) | 93 (82.3) | 0.092 |
| Yes | 57 (21.3) | 20 (17.7) |  |
| Heart disease, n (%): No | 227 (85.0) | 96 (85.0) | 0.002 |
| Yes | 40 (15.0) | 17 (15.0) |  |
| Dyslipidemia, n (%): No | 240 (89.9) | 98 (86.7) | 0.099 |
| Yes | 27 (10.1) | 15 (13.3) |  |
| Fracture-type code, n (%): Code 1 | 148 (55.4) | 58 (51.3) | 0.082 |
| Code 2 | 119 (44.6) | 55 (48.7) |  |
| QDBS, n (%): No | 150 (56.2) | 59 (52.2) | 0.080 |
| Yes | 117 (43.8) | 54 (47.8) |  |
| QSBS, n (%): No | 169 (63.3) | 59 (52.2) | 0.226 |
| Yes | 98 (36.7) | 54 (47.8) |  |
| CCBS, n (%): No | 203 (76.0) | 83 (73.5) | 0.059 |
| Yes | 64 (24.0) | 30 (26.5) |  |
| PBSB, n (%): No | 127 (47.6) | 53 (46.9) | 0.013 |
| Yes | 140 (52.4) | 60 (53.1) |  |
| YDBS, n (%): No | 141 (52.8) | 47 (41.6) | 0.226 |
| Yes | 126 (47.2) | 66 (58.4) |  |
| DHBSO, n (%): No | 126 (47.2) | 61 (54.0) | 0.136 |
| Yes | 141 (52.8) | 52 (46.0) |  |
| HTBS, n (%): No | 150 (56.2) | 58 (51.3) | 0.097 |
| Yes | 117 (43.8) | 55 (48.7) |  |
| Age, median [IQR] | 80.00 [72.00, 86.00] | 77.00 [69.00, 85.00] | 0.181 |
| Height, median [IQR] | 1.58 [1.55, 1.65] | 1.60 [1.55, 1.65] | 0.147 |
| Weight, median [IQR] | 54.00 [45.00, 60.00] | 56.00 [45.00, 61.00] | <0.001 |
| BMI, median [IQR] | 20.81 [18.11, 24.22] | 21.48 [18.14, 24.39] | 0.066 |
| Injury-to-surgery interval, median [IQR] | 144.00 [87.00, 235.00] | 103.00 [73.00, 192.00] | 0.112 |
| Immobilization time, median [IQR] | 80.00 [60.00, 103.50] | 70.00 [60.00, 97.00] | 0.046 |
| PT, median [IQR] | 11.10 [10.60, 11.50] | 11.10 [10.70, 11.80] | 0.081 |
| INR, median [IQR] | 0.92 [0.88, 0.96] | 0.92 [0.89, 0.98] | 0.060 |
| APTT, median [IQR] | 24.60 [22.20, 28.80] | 25.70 [23.30, 31.40] | <0.001 |
| Fibrinogen, median [IQR] | 4.31 [3.33, 5.30] | 4.22 [3.59, 5.12] | 0.066 |
| D-dimer, median [IQR] | 6.42 [2.97, 14.84] | 7.93 [3.91, 16.42] | 0.119 |
| CRP, median [IQR] | 50.10 [23.20, 82.45] | 38.60 [17.00, 76.40] | 0.257 |
| FBG, median [IQR] | 6.24 [5.71, 7.47] | 6.07 [5.56, 7.04] | 0.241 |
| TG, median [IQR] | 1.04 [0.81, 1.44] | 0.96 [0.78, 1.30] | 0.101 |
| TyG, median [IQR] | 8.61 [8.29, 8.95] | 8.53 [8.30, 8.82] | 0.181 |
| TC, median [IQR] | 4.29 [3.51, 5.08] | 4.24 [3.76, 4.79] | 0.042 |
| HDL-C, median [IQR] | 1.23 [1.03, 1.45] | 1.25 [1.06, 1.48] | 0.034 |
| LDL-C, median [IQR] | 2.68 [1.98, 3.26] | 2.66 [2.12, 3.18] | 0.070 |
| WBC, median [IQR] | 8.91 [7.26, 10.38] | 8.83 [7.53, 10.47] | 0.075 |
| Neutrophils, median [IQR] | 6.94 [5.34, 8.22] | 7.18 [5.60, 8.10] | 0.017 |
| Lymphocytes, median [IQR] | 1.17 [0.88, 1.56] | 1.25 [0.96, 1.50] | 0.141 |
| RBC, median [IQR] | 3.66 [3.11, 4.15] | 3.96 [3.52, 4.20] | 0.313 |
| Hemoglobin, median [IQR] | 113.00 [95.00, 127.00] | 118.00 [102.00, 130.00] | 0.124 |
| Platelet count, median [IQR] | 223.00 [188.00, 277.50] | 218.00 [180.00, 266.00] | 0.047 |

*Note: Exact-profile groups were kept intact during partitioning. DVT incidence was 27.7% in Development and 27.4% in Validation. Residual imbalance was retained rather than breaking profile groups across partitions.*

**Supplementary Table S2. Pairwise DeLong comparisons among the nine models in the profile-disjoint validation set.**

| **Model 1** | **Model 2** | **AUC 1** | **AUC 2** | **ΔAUC (1−2)** | **DeLong p** | **FDR-BH p** |
| --- | --- | --- | --- | --- | --- | --- |
| Logistic regression | SVM | 0.839 | 0.873 | -0.034 | 0.119 | 0.238 |
| Logistic regression | KNN | 0.839 | 0.622 | 0.217 | <0.001 | <0.001 |
| Logistic regression | Neural network | 0.839 | 0.810 | 0.029 | 0.068 | 0.164 |
| Logistic regression | Random forest | 0.839 | 0.859 | -0.019 | 0.562 | 0.652 |
| Logistic regression | GBM | 0.839 | 0.868 | -0.029 | 0.282 | 0.407 |
| Logistic regression | AdaBoost | 0.839 | 0.782 | 0.057 | 0.159 | 0.277 |
| Logistic regression | XGBoost | 0.839 | 0.852 | -0.013 | 0.692 | 0.754 |
| Logistic regression | LightGBM | 0.839 | 0.830 | 0.009 | 0.797 | 0.820 |
| SVM | KNN | 0.873 | 0.622 | 0.251 | <0.001 | <0.001 |
| SVM | Neural network | 0.873 | 0.810 | 0.063 | 0.052 | 0.144 |
| SVM | Random forest | 0.873 | 0.859 | 0.014 | 0.532 | 0.644 |
| SVM | GBM | 0.873 | 0.868 | 0.005 | 0.822 | 0.822 |
| SVM | AdaBoost | 0.873 | 0.782 | 0.091 | 0.014 | 0.042 |
| SVM | XGBoost | 0.873 | 0.852 | 0.021 | 0.444 | 0.591 |
| SVM | LightGBM | 0.873 | 0.830 | 0.043 | 0.146 | 0.277 |
| KNN | Neural network | 0.622 | 0.810 | -0.188 | 0.001 | 0.007 |
| KNN | Random forest | 0.622 | 0.859 | -0.236 | <0.001 | <0.001 |
| KNN | GBM | 0.622 | 0.868 | -0.246 | <0.001 | <0.001 |
| KNN | AdaBoost | 0.622 | 0.782 | -0.160 | 0.010 | 0.032 |
| KNN | XGBoost | 0.622 | 0.852 | -0.230 | <0.001 | <0.001 |
| KNN | LightGBM | 0.622 | 0.830 | -0.208 | <0.001 | 0.002 |
| Neural network | Random forest | 0.810 | 0.859 | -0.049 | 0.219 | 0.343 |
| Neural network | GBM | 0.810 | 0.868 | -0.058 | 0.080 | 0.180 |
| Neural network | AdaBoost | 0.810 | 0.782 | 0.028 | 0.537 | 0.644 |
| Neural network | XGBoost | 0.810 | 0.852 | -0.042 | 0.272 | 0.407 |
| Neural network | LightGBM | 0.810 | 0.830 | -0.020 | 0.620 | 0.697 |
| Random forest | GBM | 0.859 | 0.868 | -0.010 | 0.475 | 0.610 |
| Random forest | AdaBoost | 0.859 | 0.782 | 0.077 | 0.003 | 0.011 |
| Random forest | XGBoost | 0.859 | 0.852 | 0.006 | 0.736 | 0.779 |
| Random forest | LightGBM | 0.859 | 0.830 | 0.029 | 0.161 | 0.277 |
| GBM | AdaBoost | 0.868 | 0.782 | 0.087 | <0.001 | 0.003 |
| GBM | XGBoost | 0.868 | 0.852 | 0.016 | 0.348 | 0.481 |
| GBM | LightGBM | 0.868 | 0.830 | 0.038 | 0.063 | 0.163 |
| AdaBoost | XGBoost | 0.782 | 0.852 | -0.070 | 0.009 | 0.032 |
| AdaBoost | LightGBM | 0.782 | 0.830 | -0.048 | 0.116 | 0.238 |
| XGBoost | LightGBM | 0.852 | 0.830 | 0.022 | 0.207 | 0.339 |

*Note: All comparisons used the same profile-disjoint validation set (n=113; 31 DVT events). FDR-BH p values are provided for the 36 pairwise comparisons.*

**Supplementary Table S3A. Pairwise phi correlations among the seven TCM syndrome indicators.**

| **Variable** | **QDBS** | **QSBS** | **YDBS** | **CCBS** | **DHBSO** | **PBSB** | **HTBS** |
| --- | --- | --- | --- | --- | --- | --- | --- |
| QDBS | 1.000 | 0.266 | 0.038 | 0.058 | 0.086 | 0.074 | -0.047 |
| QSBS | 0.266 | 1.000 | 0.142 | -0.032 | 0.095 | 0.022 | 0.002 |
| YDBS | 0.038 | 0.142 | 1.000 | -0.030 | 0.089 | -0.032 | 0.022 |
| CCBS | 0.058 | -0.032 | -0.030 | 1.000 | 0.015 | 0.104 | 0.091 |
| DHBSO | 0.086 | 0.095 | 0.089 | 0.015 | 1.000 | 0.004 | -0.046 |
| PBSB | 0.074 | 0.022 | -0.032 | 0.104 | 0.004 | 1.000 | -0.059 |
| HTBS | -0.047 | 0.002 | 0.022 | 0.091 | -0.046 | -0.059 | 1.000 |

**Supplementary Table S3B. Variance-inflation factors for the seven TCM syndrome indicators.**

| **TCM syndrome** | **VIF** |
| --- | --- |
| QDBS | 1.092 |
| QSBS | 1.104 |
| YDBS | 1.029 |
| CCBS | 1.028 |
| DHBSO | 1.022 |
| PBSB | 1.021 |
| HTBS | 1.019 |

*Note: Pairwise phi coefficients were small to moderate and all VIFs were close to 1, providing no evidence of problematic collinearity among the seven syndrome indicators.*

**Supplementary Table S4. Incremental predictive value of QDBS and QSBS.**

| **Metric** | **Clinical/laboratory RF** | **Integrated RF** | **Difference / estimate** | **95% CI** | **Statistical test** |
| --- | --- | --- | --- | --- | --- |
| AUC | 0.855 | 0.859 | ΔAUC +0.003 |  | DeLong p=0.746 |
| Brier score | 0.134 | 0.132 | Δ -0.002 |  |  |
| Calibration intercept | -0.131 | -0.305 |  |  |  |
| Calibration slope | 1.163 | 1.136 |  |  |  |
| Continuous NRI |  |  | 0.621 | 0.263–0.943 |  |
| IDI |  |  | 0.035 | 0.017–0.053 |  |

*Note: The clinical/laboratory RF used D-dimer, fibrinogen, platelet count, T2D, FBG, and TyG. The integrated RF additionally included QSBS and QDBS. The AUC gain was minimal and non-significant; NRI and IDI were secondary reclassification measures.*

**Supplementary Table S5. Primary no-SMOTE RF analysis versus fold-nested SMOTENC sensitivity analysis.**

| **Metric** | **Primary no-SMOTE** | **Fold-nested SMOTENC** | **Difference** | **Statistical test** |
| --- | --- | --- | --- | --- |
| Development OOF AUC | 0.774 | 0.763 |  |  |
| Validation AUC | 0.859 | 0.859 | +0.0006 | Paired DeLong p=0.985 |
| Brier score | 0.132 | 0.158 |  |  |
| Calibration slope | 1.136 | 1.199 |  |  |

*Note: SMOTENC was applied only within development resampling. All validation metrics were calculated in the original non-augmented held-out validation set.*

**Supplementary Table S6A. Thirty repeated profile-aware development-validation partitions.**

| **Repeat** | **Status** | **Validation n** | **Validation events** | **Profile overlap** | **AUC** |
| --- | --- | --- | --- | --- | --- |
| 1 | OK | 113 | 31 | 0 | 0.844 |
| 2 | OK | 113 | 31 | 0 | 0.821 |
| 3 | OK | 113 | 31 | 0 | 0.863 |
| 4 | OK | 113 | 31 | 0 | 0.719 |
| 5 | OK | 113 | 31 | 0 | 0.844 |
| 6 | OK | 113 | 31 | 0 | 0.804 |
| 7 | OK | 113 | 31 | 0 | 0.799 |
| 8 | OK | 113 | 31 | 0 | 0.749 |
| 9 | OK | 113 | 31 | 0 | 0.775 |
| 10 | OK | 113 | 31 | 0 | 0.763 |
| 11 | OK | 113 | 31 | 0 | 0.775 |
| 12 | OK | 113 | 31 | 0 | 0.803 |
| 13 | OK | 113 | 31 | 0 | 0.753 |
| 14 | OK | 113 | 31 | 0 | 0.889 |
| 15 | OK | 113 | 31 | 0 | 0.798 |
| 16 | OK | 113 | 31 | 0 | 0.730 |
| 17 | OK | 113 | 31 | 0 | 0.833 |
| 18 | OK | 113 | 31 | 0 | 0.770 |
| 19 | OK | 113 | 31 | 0 | 0.773 |
| 20 | OK | 113 | 31 | 0 | 0.847 |
| 21 | OK | 113 | 31 | 0 | 0.812 |
| 22 | OK | 113 | 31 | 0 | 0.748 |
| 23 | OK | 113 | 31 | 0 | 0.814 |
| 24 | OK | 113 | 31 | 0 | 0.869 |
| 25 | OK | 113 | 31 | 0 | 0.792 |
| 26 | OK | 113 | 31 | 0 | 0.654 |
| 27 | OK | 113 | 31 | 0 | 0.742 |
| 28 | OK | 113 | 31 | 0 | 0.758 |
| 29 | OK | 113 | 31 | 0 | 0.887 |
| 30 | OK | 113 | 31 | 0 | 0.747 |

**Supplementary Table S6B. Summary of the 30 repeated profile-aware RF AUCs.**

| **Model** | **Successful repeats** | **Mean AUC** | **SD** | **Median AUC** | **2.5th–97.5th percentile** | **Min–max** |
| --- | --- | --- | --- | --- | --- | --- |
| Random forest | 30 | 0.792 | 0.054 | 0.795 | 0.701–0.887 | 0.654–0.889 |

*Note: All 30 repeated partitions retained exact-profile groups intact and had zero development-validation profile overlap. The percentile interval summarizes split-to-split variability and is not an independent-sample confidence interval.*

**Supplementary Table S7. Exploratory TyG interaction analyses with likelihood-ratio testing and FDR correction.**

| **Modifier** | **Level** | **N** | **Events** | **OR per 1-SD TyG** | **95% CI** | **P-interaction** | **FDR p** |
| --- | --- | --- | --- | --- | --- | --- | --- |
| Sex | Female | 268 | 86 | 1.806 | 1.388–2.350 | 0.0056 | 0.033 |
| Sex | Male | 112 | 19 | 0.681 | 0.353–1.315 | 0.0056 | 0.033 |
| Age group | <79 years | 207 | 57 | 1.515 | 1.137–2.018 | 0.357 | 0.620 |
| Age group | ≥79 years | 173 | 48 | 1.901 | 1.285–2.813 | 0.357 | 0.620 |
| Type 2 diabetes | No | 281 | 63 | 1.633 | 1.219–2.187 | 0.413 | 0.620 |
| Type 2 diabetes | Yes | 99 | 42 | 1.333 | 0.906–1.961 | 0.413 | 0.620 |
| Prior stroke | No | 303 | 71 | 1.695 | 1.277–2.250 | 0.545 | 0.654 |
| Prior stroke | Yes | 77 | 34 | 1.442 | 0.938–2.219 | 0.545 | 0.654 |
| QDBS | No | 209 | 48 | 1.693 | 1.252–2.289 | 0.729 | 0.729 |
| QDBS | Yes | 171 | 57 | 1.557 | 1.082–2.242 | 0.729 | 0.729 |
| QSBS | No | 228 | 54 | 1.879 | 1.379–2.559 | 0.310 | 0.620 |
| QSBS | Yes | 152 | 51 | 1.458 | 1.005–2.116 | 0.310 | 0.620 |

*Note: Each interaction model contained TyG (per 1 SD), the subgroup variable, and their interaction term without additional covariate adjustment. The age cutoff was the development-set median (79 years). These analyses were exploratory.*

**Supplementary Table S8A. Descriptive RF subgroup discrimination and classification performance.**

| **Subgroup** | **Level** | **N** | **Events** | **AUC** | **Sensitivity** | **Specificity** | **PPV** | **NPV** |
| --- | --- | --- | --- | --- | --- | --- | --- | --- |
| Sex | Female | 81 | 26 | 0.806 | 0.885 | 0.618 | 0.523 | 0.919 |
| Sex | Male | 32 | 5 | 1.000 | 1.000 | 0.926 | 0.714 | 1.000 |
| Age group | <79 years | 74 | 19 | 0.907 | 1.000 | 0.764 | 0.594 | 1.000 |
| Age group | ≥79 years | 39 | 12 | 0.796 | 0.750 | 0.630 | 0.474 | 0.850 |

**Supplementary Table S8B. Descriptive RF subgroup calibration.**

| **Subgroup** | **Level** | **N** | **Events** | **Calibration intercept** | **Calibration slope** |
| --- | --- | --- | --- | --- | --- |
| Sex | Female | 81 | 26 | -0.303 | 0.883 |
| Sex | Male | 32 | 5 |  |  |
| Age group | <79 years | 74 | 19 | 0.042 | 1.847 |
| Age group | ≥79 years | 39 | 12 | -0.521 | 0.714 |

*Note: These subgroup estimates are descriptive internal-validation results and should not be interpreted as evidence of fairness or subgroup-specific clinical performance. Calibration parameters were not estimated in the male subgroup because only five validation events were present.*

**Supplementary Table S9. Missingness and imputation audit.**

| **Variable** | **Missing n** | **Missing (%)** |
| --- | --- | --- |
| DVT outcome | 0 | 0.0 |
| Sex | 0 | 0.0 |
| Hypertension | 0 | 0.0 |
| Type 2 diabetes | 0 | 0.0 |
| Prior stroke | 0 | 0.0 |
| Heart disease | 0 | 0.0 |
| Dyslipidemia | 0 | 0.0 |
| QDBS | 0 | 0.0 |
| QSBS | 0 | 0.0 |
| CCBS | 0 | 0.0 |
| PBSB | 0 | 0.0 |
| YDBS | 0 | 0.0 |
| DHBSO | 0 | 0.0 |
| HTBS | 0 | 0.0 |
| Age | 0 | 0.0 |
| Height | 0 | 0.0 |
| Weight | 0 | 0.0 |
| BMI | 0 | 0.0 |
| Injury-to-surgery interval | 0 | 0.0 |
| Immobilization time | 0 | 0.0 |
| PT | 0 | 0.0 |
| INR | 0 | 0.0 |
| APTT | 0 | 0.0 |
| Fibrinogen | 0 | 0.0 |
| D-dimer | 0 | 0.0 |
| CRP | 0 | 0.0 |
| FBG | 0 | 0.0 |
| TG | 0 | 0.0 |
| TyG | 0 | 0.0 |
| TC | 0 | 0.0 |
| HDL-C | 0 | 0.0 |
| LDL-C | 0 | 0.0 |
| WBC | 0 | 0.0 |
| Neutrophils | 0 | 0.0 |
| Lymphocytes | 0 | 0.0 |
| RBC | 0 | 0.0 |
| Hemoglobin | 0 | 0.0 |
| Platelet count | 0 | 0.0 |

*Note: No imputation was required in the final analytical cohort for the variables retained in the final analysis. The earlier exclusion of 48 records for missing key baseline variables occurred before formation of the final 380-patient cohort.*

**Supplementary Table S10. Model engines, selected hyperparameters, and reproducibility specifications.**

| **Model** | **Engine / package** | **Selected hyperparameters** | **Role** |
| --- | --- | --- | --- |
| Logistic regression | caret/MASS; caret 7.0.1; MASS 7.3.65 | parameter=none | Evaluation-only comparator |
| SVM | caret/kernlab; caret 7.0.1; kernlab 0.9.33 | sigma=0.005; C=0.5 | Evaluation-only comparator |
| KNN | caret/kknn; caret 7.0.1; kknn 1.4.1 | kmax=9; distance=1; kernel=optimal | Evaluation-only comparator |
| Neural network | caret/nnet; caret 7.0.1; nnet 7.3.20 | size=5; decay=0.5 | Evaluation-only comparator |
| Random forest | caret/ranger; caret 7.0.1; ranger 0.18.0 | mtry=4; splitrule=gini; min.node.size=1 | Index model |
| GBM | caret/gbm; caret 7.0.1; gbm 2.3.1 | n.trees=100; interaction.depth=2; shrinkage=0.05; n.minobsinnode=5 | Evaluation-only comparator |
| AdaBoost | caret/adabag; caret 7.0.1; adabag 5.1 | mfinal=50; maxdepth=2; coeflearn=Zhu | Evaluation-only comparator |
| XGBoost | xgboost; xgboost 3.2.1.1 | nrounds=100; max_depth=2; eta=0.1; gamma=0; colsample_bytree=0.8; min_child_weight=1; subsample=0.8 | Evaluation-only comparator |
| LightGBM | lightgbm; lightgbm 4.7.0 | nrounds=60; learning_rate=0.1; num_leaves=15; min_data_in_leaf=10 | Evaluation-only comparator |

*Note: All nine algorithms used the fixed predictor panel: D-dimer, fibrinogen, platelet count, T2D, FBG, TyG, QSBS, and QDBS. Tuning used repeated 10-fold cross-validation with five repeats under exact-profile group-aware resampling. The primary analysis used no class balancing; fold-nested SMOTENC was a sensitivity analysis. The full R/package environment is recorded in the supplied sessionInfo file.*

**Supplementary Figure S1. Precision-recall curves for all nine models in the profile-disjoint validation set.**

*Note: Curves are based on the common held-out validation set (n=113; 31 DVT events). The no-skill reference corresponds to the validation-set event prevalence (27.4%).*
